# The public’s preferred level of involvement in local policy-making

**DOI:** 10.1038/s41598-023-34282-w

**Published:** 2023-05-02

**Authors:** Tessa Haesevoets, Arne Roets, Ruben Van Severen, Kim Dierckx, Bram Verschuere

**Affiliations:** 1 Department of Developmental, Personality and Social Psychology, Ghent University, Henri Dunantlaan 2, 9000 Ghent, Belgium; 2 Department of Public Governance and Management, Ghent University, Ghent, Belgium

**Keywords:** Psychology, Human behaviour

## Abstract

We investigated what people consider the optimal level of citizen involvement in local policy decision-making. This is an important question to answer, given that civil servants and politicians are increasingly confronted with the pressure to add a participatory layer to representative democratic policy-making. Across five empirical studies (total *N* = 1470), we consistently found that, overall, the most preferred decision-making model is a balanced model in which citizens and the government are equally involved. Despite this preferred ‘overall’ pattern of equal involvement, we identified three subgroups within the citizenry with different preference curves: Some citizens prefer a model in which citizens and the government are truly equal partners, whereas others prefer a model in which either the government or citizens are relatively more involved in the policy decision-making process. The main contribution of our work is thus that we identified a perceived ‘overall’ optimal level of citizen engagement, and variations to that optimum depending on citizens’ individual traits. This information might be helpful to policy-makers in developing effective citizen participation processes.

## Introduction

Most of today’s democracies are representative in nature, meaning that citizens elect officials to make policy decisions, formulate laws, and administer programs for the public good. Over the last few decades, however, citizens’ confidence in and satisfaction with representative institutions has eroded^1,2^. Declining voter turnout, membership of political parties, and trust in institutions all signal that traditional representative model is under pressure^3–5^. This is expressed through growing and systematic anti-politics: Disenchantment with politics, politicians, and political parties is what primarily accounts for the decline of public engagement in traditional forms of political organization^3,6^.

### The changing nature of local democracy

To overcome these challenges, and in an attempt to reverse the declining satisfaction with representative democracy, an increasing number of (local) governments worldwide started to experiment with democratic innovations. As Bengtsson (^7^, p. 46) states: “The most commonly suggested action in order to overcome this challenge has been to bring people back into politics with the use of more participatory forms of democracy.” Participation materializes in practices like mini-publics, citizen juries, referenda, participatory budgeting and the like, which all have in common that they add a participatory layer to representative democracy, by enabling citizens to participate more directly in policy decisions^8–10^.

Empirical evidence gleaned from public opinion polls demonstrates that most citizens are supportive of a more participatory version of democracy (e.g.,^11–13^). Dalton et al. (^12^, p. 145), for instance, state that: “most people in Western democracies favor reforms that would move toward a more participatory style of democratic government.” Along similar lines, Bowler et al. (^11^, p. 351) noted that: “most people surveyed in affluent democracies appear to demand, or at least approve of, direct citizen influence over policy decisions.” A survey conducted by Pew Research Center among 41,953 respondents from 38 different countries confirmed the findings mentioned above, by illustrating that direct democracy is supported by roughly two-thirds of the public^14^; with little difference in views between different countries.

Despite this undeniable trend towards more direct citizen participation, and its popularity worldwide^11,14,15^, it remains unclear how much input ordinary people prefer citizens to have—relative to the government—in (local) policy-making processes. This question is critical, given that between purely representative and purely direct democratic models, in practice, there is room for many “intermediate models” (^16^, p. 162), with varying degrees of citizen and government involvement. However, although we have some evidence of the legitimacy of direct democracy in the eyes of the citizenry (cf. supra), to date, we do not know how much input people actually prefer citizens to have in the policy decision-making process, and how much decisional power they prefer to remain in the hands of elected politicians. Neither do we know whether there are differences within the citizenry (i.e., subgroups) with regard to the perceived optimal level of citizen involvement, and what may characterize these subgroups.

The answer to these questions does not only have theoretical relevance, but knowledge about the perceived optimal level of citizen involvement—and potential individual differences therein—may also be useful for politicians and civil servants, who are tasked with developing effective citizen participation initiatives in practice, and for whom it may not always be clear how much say citizens exactly want to have in such initiatives. Our findings might provide them some insights into the public’s preferred level of involvement in policy-making.

### Research objectives

The main aim of the present study is to analyze in detail how the general public believes decisional power in policy-making should be balanced between citizens (participation) and their local government (representation). More specifically, our research goes beyond existing studies by employing a rigorous ‘multi-study, multi-method’ approach to empirically test which particular mixture of citizen and government involvement people consider to be optimal in the context of local policy-making scenarios. The objectives of our research are threefold.

#### Objective 1: measuring the preferred level of citizen involvement

The first objective of our research is to examine people’s general conceptions of how the balance of power in policy decisions should ideally look like. On a general level, do people want citizens to completely take over decisional power from elected officials? Or do they prefer citizens and the government to both have a say in policy decisions, but with specific relative weights? In light of these questions, we argue that recent calls for greater citizen involvement reflect a general desire for more direct citizen participation in policy-making processes, but not necessarily a desire for complete citizen control. To pinpoint the optimal level of desired citizen involvement, as a first research objective, we investigated how much decisional weight ordinary people prefer citizens and the government to have—relative to each other—in the context of local policy decision-making scenarios. The local level is often described by political theorists as an ideal arena for the empowerment of citizens and as an important learning school for democracy, and is therefore considered particularly suited to study new forms of citizen engagement (^17,18^; for two concrete examples of studies examining citizen participation at the local level, see^19,20^).

#### Objective 2: identifying clusters in the population based on preference patterns

Even though we expect that, overall, people prefer a model in which citizens and the government both have a considerable weight in local policy decisions, different patterns in what is considered the optimal distribution of decision power are likely to exist in the general population. We therefore also examined whether different subgroups (i.e., clusters) of the citizenry can be identified, who react differently to increasing levels of citizen involvement. Although citizens’ preferences for different decision-making models were “left untouched for a long time” (^21^, p. 235), some recent attempts to expand knowledge on this topic have been made. These studies found that some people favour high levels of citizen involvement, while others are more supportive of leaving decisions in the hands of politicians or expects (e.g.,^11,22–25^). Critically, however, is that this prior work did not investigate how people respond to increasing levels of citizen (vs. government) involvement, neither did it examine what people consider the optimal mixture of citizen and government involvement.

Which subgroups then might exist? We predict that there is a first subgroup of people who have a preference for the government as main decision-maker, and who are thus expected to react negatively to increasing levels of citizen involvement. Conversely, we also envision a second subgroup of people who prefer citizens as main decision-makers, and who thus react positively to increasing levels of citizens involvement. Additionally, we hypothesize that there might also be a third subgroup who prefers a more balanced model: These people are expected to react positively to increasing levels of citizen involvement, but only up to a certain point after which additional involvement from citizens is expected to elicit negative reactions. Our second research objective is thus to empirically establish which subgroups exist within the citizenry, examine how large the emerging subgroups are, and pinpoint where the optimal level of desired citizen involvement is located within each of the subgroups.

#### Objective 3: defining clusters in terms of individual traits

Although some recent scholarly attention has been dedicated to people’s preferences for citizen involvement in policy decision-making, only few efforts were made to identify who these people are and why they display a certain preference^21,25^. Besides classical demographic or socio-economic variables, these prior studies mainly focused on variables like political dissatisfaction and political or civil engagement, but did not pay much attention to a broader array of individual traits and personality dimensions that may also be relevant to determine people’s preferences. To address this gap, as a third research objective, we explored if robust trait and personality differences exist between the clusters that can be empirically distinguished within the citizenry.

A first category of individual traits—which may be particularly relevant in the context of our study—are Right-Wing Authoritarianism (RWA), Left-Wing Authoritarianism (LWA), and Social Dominance Orientation (SDO). RWA reflects social-cultural right-wing beliefs. People who score high on RWA are submissive to authority figures, act aggressively in the name of authorities, and are conventional in thought and behaviour^26–28^. LWA, on the other hand, describes authoritarianism in service of left-wing outcomes. LWA resembles RWA in being characterized by high levels of dogmatism. What distinguishes these two forms of authoritarianism is the content of the dogmatically defended values: For individuals high in LWA these values reflect negative and punitive attitudes towards advantaged groups, a desire to (violently) break the status quo, and impose new (left-wing) rules on society^29,30^. SDO, in turn, is a typical indicator of economic hierarchical right-wing beliefs. High scorers on this trait generally prefer intergroup and interpersonal relations to be hierarchical, and tend to favour and maintain policies that preserve social inequality^31^. In light of these ideological attitudes, it can thus be expected that if a cluster of citizens emerges who prefer the government to be the main decision-maker, this subgroup will score relatively high on RWA and SDO and lower on LWA.

Another potentially relevant category of individual traits concerns political and social cynicism. A cynical person is someone who shows a disposition to disbelieve in the sincerity or goodness of human motives and actions. Political and social cynics differ in the extent to which they generalize their cynical attitudes: Whereas social cynics question the motives of nearly all humans, political cynics’ negative attitudes are specifically directed towards politicians^32^. As such, if a cluster of citizens emerges who prefer citizens as main decision-makers, this subgroup can then be expected to show relatively high levels of political cynicism but lower levels of social cynicism. For exploratory purposes, we additionally also tested if the subgroups differ in terms of the HEXACO personality dimensions^33^. Given our exploratory purposes, we had no specific predictions regarding how emerging clusters would differ in terms of these personality dimensions.

## The present studies

The three aforementioned research objectives were tested across five empirical studies. Table 1 summarizes the main characteristic of our five studies. As shown in this table, we started our research endeavour with an explorative study (Study 1), which aimed to obtain a general estimate of the desired relative weight of citizen and government involvement in policy decisions (Objective 1). This explorative study was supplemented with four additional studies (Studies 2–5), in which we used a variety of different research methods and designs (see Table 1 for an overview) to map what particular combination of citizen and government involvement at the local level people consider to be optimal (Objective 1). Studies 3, 4, and 5 additionally also examined the existence of possible clusters within the citizenry based on their preferences (Objective 2), with Studies 4 and 5 also exploring the defining personality characteristics of these clusters (Objective 3). Sensitivity power analyses using the WebPower package^34^ and the simr package^35^ in R showed that our studies were all sufficiently powered to detect the reported effects (see Appendix A for more details). Unless mentioned otherwise, the data of our studies were analyzed using SPSS (version 27). The datasets and data analysis scripts are publicly available at: https://osf.io/zs9cj/.

**Table 1:** Summary of the main characteristics of the five empirical studies.

Study	*N*	Research design	Context	Presentation order	Test of objective(s)
Study 1	200	Constant-sum scale	Abstract	–	Objective 1
Study 2	270	Between-subjects design	Abstract	–	Objective 1
Study 3	294	Within-subjects design	Abstract	Fixed	Objectives 1 & 2
Study 4	409	Mixed-factorial design	Concrete	Random	Objectives 1, 2 & 3
Study 5	297	Pairwise comparisons	Abstract	Random	Objectives 1, 2 & 3

The ‘Context’ variable reflects whether the situation presented to participants concerns an abstract (general) or a concrete (specific) case. The ‘Presentation Order’ variable reflects whether the decision-making models (in Studies 3 and 4) and the pairwise comparisons (in Study 5) were presented to participants in a fixed or a random order.

Participants in our studies were all recruited via Prolific (www.prolific.co), an online research platform that provides a detailed description of the demographics of their participant pool, and which can be used by researchers to a priori select specific groups of participants (for more detailed information on this platform, see^36,37^). In all five studies, we recruited a gender-balanced sample of adult participants living in the United Kingdom. No further selection criteria were used. The five samples were independent and did not overlap. The UK is a highly appropriate context for the purpose of our research, because both representative and more direct forms of democracy are already used at different levels of government^38^, including the local level. The Edinburgh Road Tolls Referendum is an example of a local referendum, held in February 2005 by the City of Edinburgh Council, about whether voters supported the Council’s proposed transport strategy^39^. An example of a local citizens’ jury is the Leeds Climate Change Citizens’ Jury, which was commissioned in 2019 by the Leeds Climate Commission to ensure citizens’ voices were heard in Leeds’ vision for achieving carbon zero emissions^40^. Finally, an example of a local participatory budgeting project is ‘You Decide!’. This project was carried out in 2009–2010 in Tower Hamlets, a dense urban Borough in the East End of London^41^. These examples illustrate that, in the last decade, various forms of direct citizen participation have been used by UK local governments.

### Study 1

Our first study (*N* = 200) was an explorative study in which participants were asked to indicate how decisional power in local policy-making should be balanced between citizens and the government. To this ends, we employed a constant-sum approach^42^, which allowed us to directly compare the relative importance that people ascribe to the direct involvement and decisional power of citizens and the local government (see “Methods” section for more details).

#### Results of study 1

**Figure 1:**
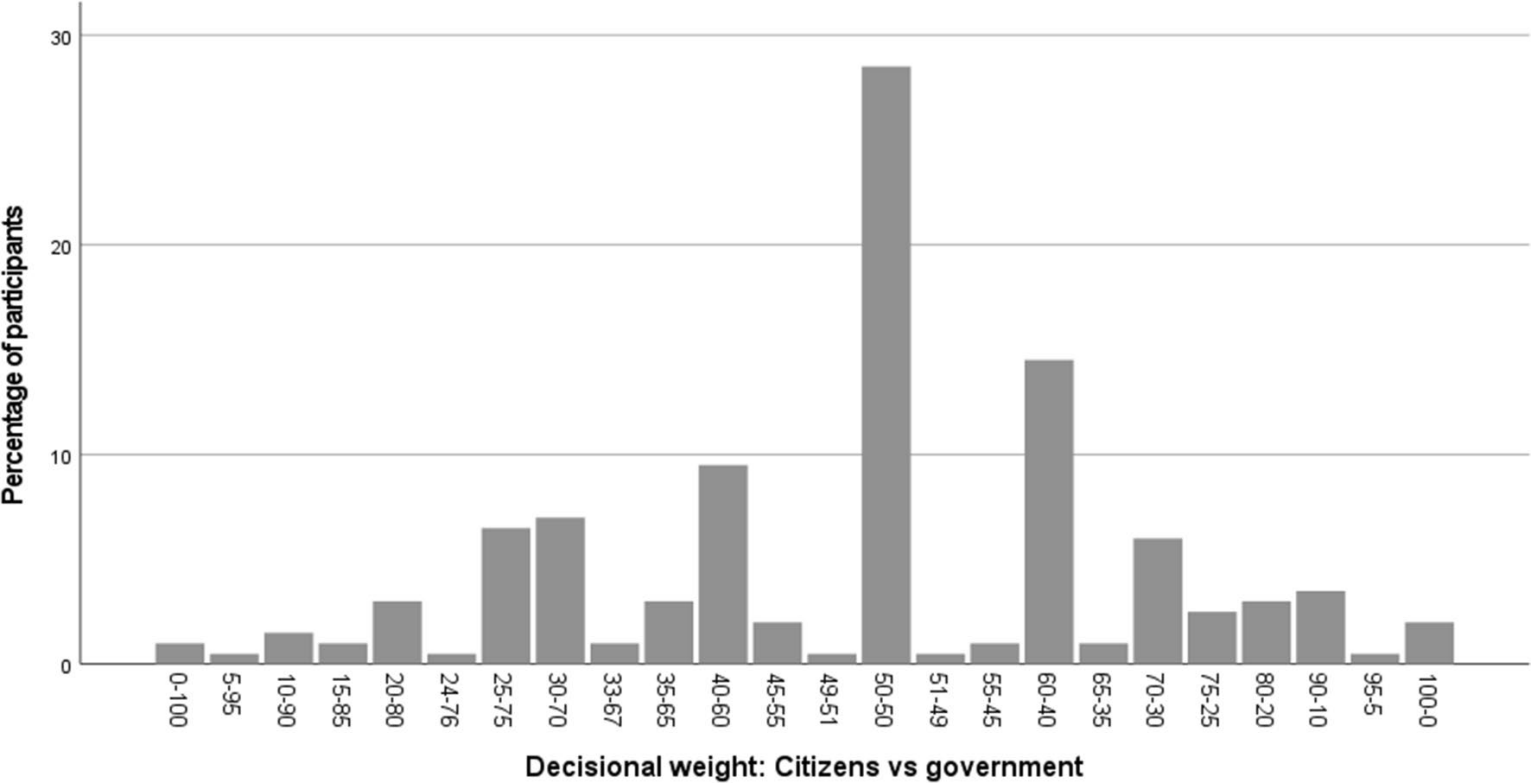
Distribution of participant’s preferred decisional weight (Study 1).

Figure 1 shows the distribution of participant’s preferred decisional weight. Our analyses revealed that, on average, participants preferred citizens to have a decisional weight of 49.6% and the local government to have a decisional weight of 50.4% (*SD* = 19.79). A closer inspection of participants’ preferences (see Fig. 1), revealed that 37.0% of the participants (74 out of 200) preferred the government to outweigh citizens. Of the remaining participants, 28.5% (57 out of 200) preferred citizens and the government to both have a weight of exactly 50%, and 34.5% (69 out of 200) preferred citizens to outweigh the government. What is particularly interesting, however, is that only a very small percentage of participants (less than 5%) preferred either the government or citizens to have full decisional control (i.e., a decisional weight of 100% for one of the two actors)—see Fig. 1. These findings hence show that most people indeed seem to prefer a model in which citizens and the government both have a considerable weight, but also that the preferred amount of citizen (vs. government) involvement varies strongly across individuals (with some preferring small and others preferring large levels of citizen involvement).

### Study 2

Our second study (*N* = 270) was an experimental study in which participants were randomly assigned to one of five conditions which varied the decisional weight that citizens have—relative to the government—in a local policy decision-making scenario (citizen vs. government weight: 0% vs. 100%, 25% vs. 75%, 50% vs. 50%, 75% vs. 25%, 100% vs. 0%). Participants were asked to indicate to what extent they found the presented decision-making model acceptable, legitimate, fair, democratic, effective, efficient, appropriate, and justified (1 = *not at all*, 10 = *very much so*).

#### Results of study 2

**Figure 2:**
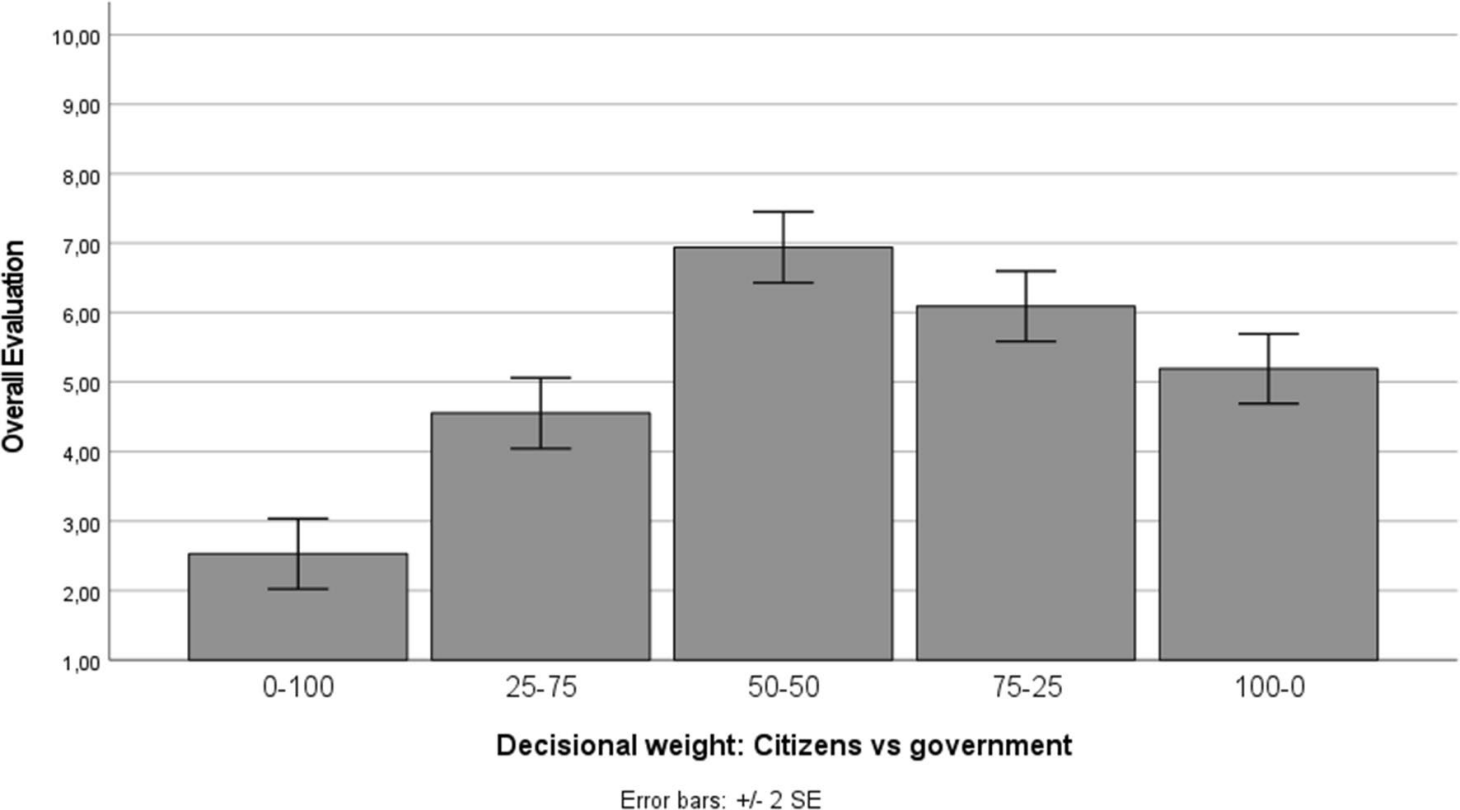
Overall evaluation (mean of acceptable, legitimate, fair, democratic, effective, efficient, appropriate, and justified) in function of the decision-making models (Study 2).

An analysis of variance (ANOVA) revealed that participants’ overall evaluation of the decision-making models (i.e., the mean of acceptable, legitimate, fair, democratic, effective, efficient, appropriate, and justified) differed significantly across the five experimental conditions, *F*(4, 265) = 43.67, *p* < 0.001, partial *η*^*2*^ = 0.397. Figure 2 displays participants’ overall evaluation of the different decision-making models. As shown in this figure, participants’ overall evaluation was highest in the condition in which citizens and the government both have a decisional weight of 50%. Post-hoc comparisons with Tukey’s honestly significant difference (HSD) correction showed that the overall evaluation in this condition differed significantly from all the other conditions (all *p*s < 0.001), with exception of the condition in which citizens have 75% and the government has 25% weight (*p* = 0.129). These findings thus indicate that there is, on average, a clear preference for models in which citizens have at least 50% weight in the decision, and although in case of unequal weight people seem to prefer citizens rather than the government to have a greater say, support decreases when citizens envision having absolute control in local policy-making processes.

### Study 3

This third study (*N* = 294) aimed to replicate and extend our prior findings using yet another research design. More specifically, we employed a similar setup as in the second study, but this time we used a within-subjects (instead of a between-subjects) design to administer the different decision-making models, which allowed us to test for the potential existence of different preference patterns. In order to be able to pinpoint more precisely where the general optimal level of desired citizen involvement is located, in this third study we included a total of eleven different decision-making models. These models ranged from 0% citizen and 100% government weight up to 100% citizen and 0% government weight, in small steps of 10% (see “Methods” for more details). For each resulting model, participants were asked to indicate the extent to which they found that particular model appropriate, justified, and acceptable (1 = *not at all*, 10 = *very much so*).

#### Results of study 3

**Figure 3:**
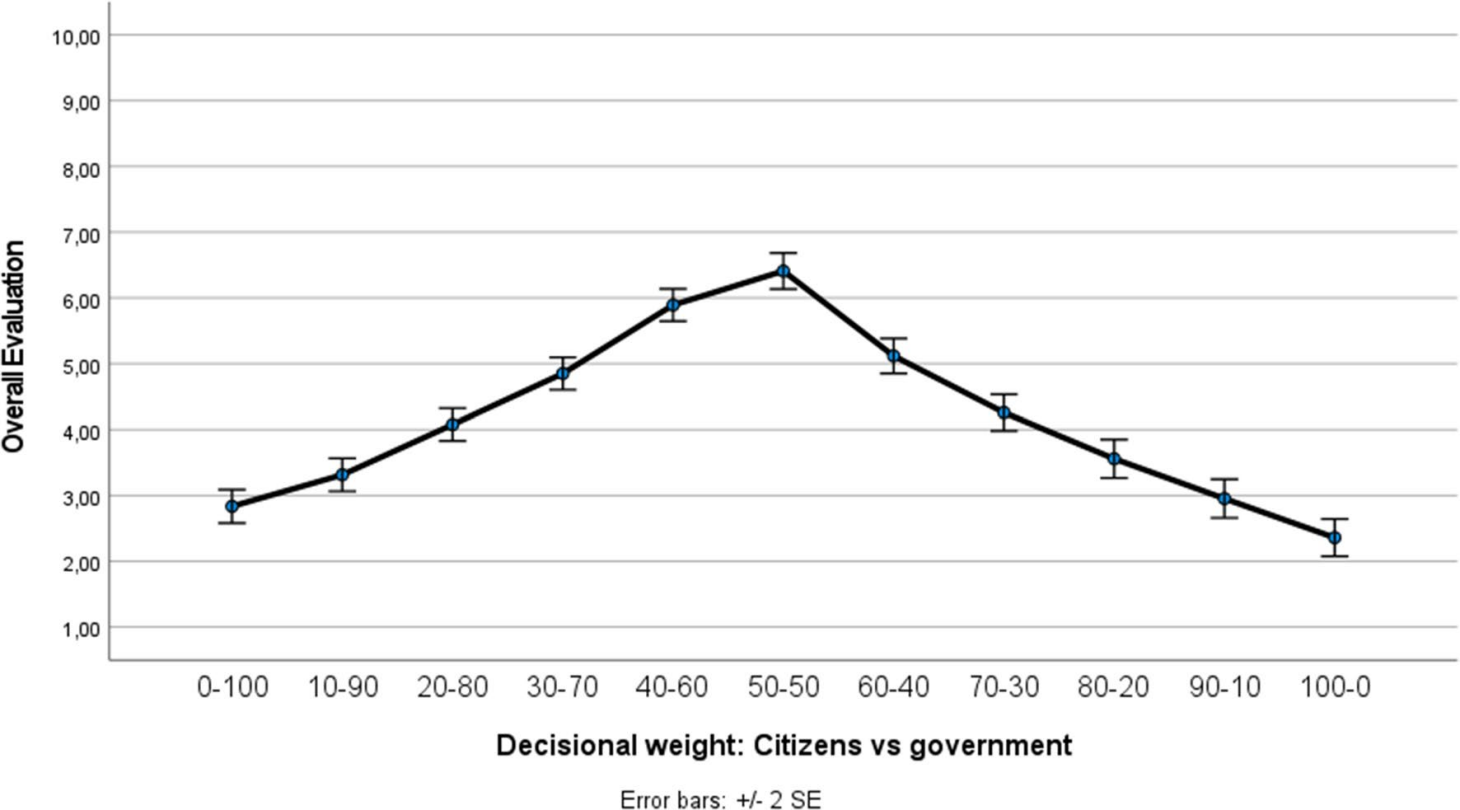
Overall evaluation (mean of appropriate, justified, and acceptable) in function of the decision-making models (Study 3).

A repeated measures ANOVA revealed that the eleven decision-making models significantly impacted participants’ overall evaluation (i.e., the mean of appropriate, justified, and acceptable), *F*(10, 284) = 71.73, *p* < 0.001, partial *η*^*2*^ = 0.716. Figure 3 visualizes how participants scored the different decision-making models. We found the overall existence of an inverted-U (or, to be more precise, an inverted-V) curve. As shown in Fig. 3, participants’ overall evaluation increased up until the point where citizens and the government both have a decisional weight of exactly 50%. After this point, higher degrees of citizen involvement negatively impacted participants’ overall evaluation. Pairwise comparisons with Sidak adjustment for multiple comparisons showed that the model in which citizens and the government both have a decisional weight of 50% differed significantly from all the other models (all *p*s < 0.046).

**Figure 4:**
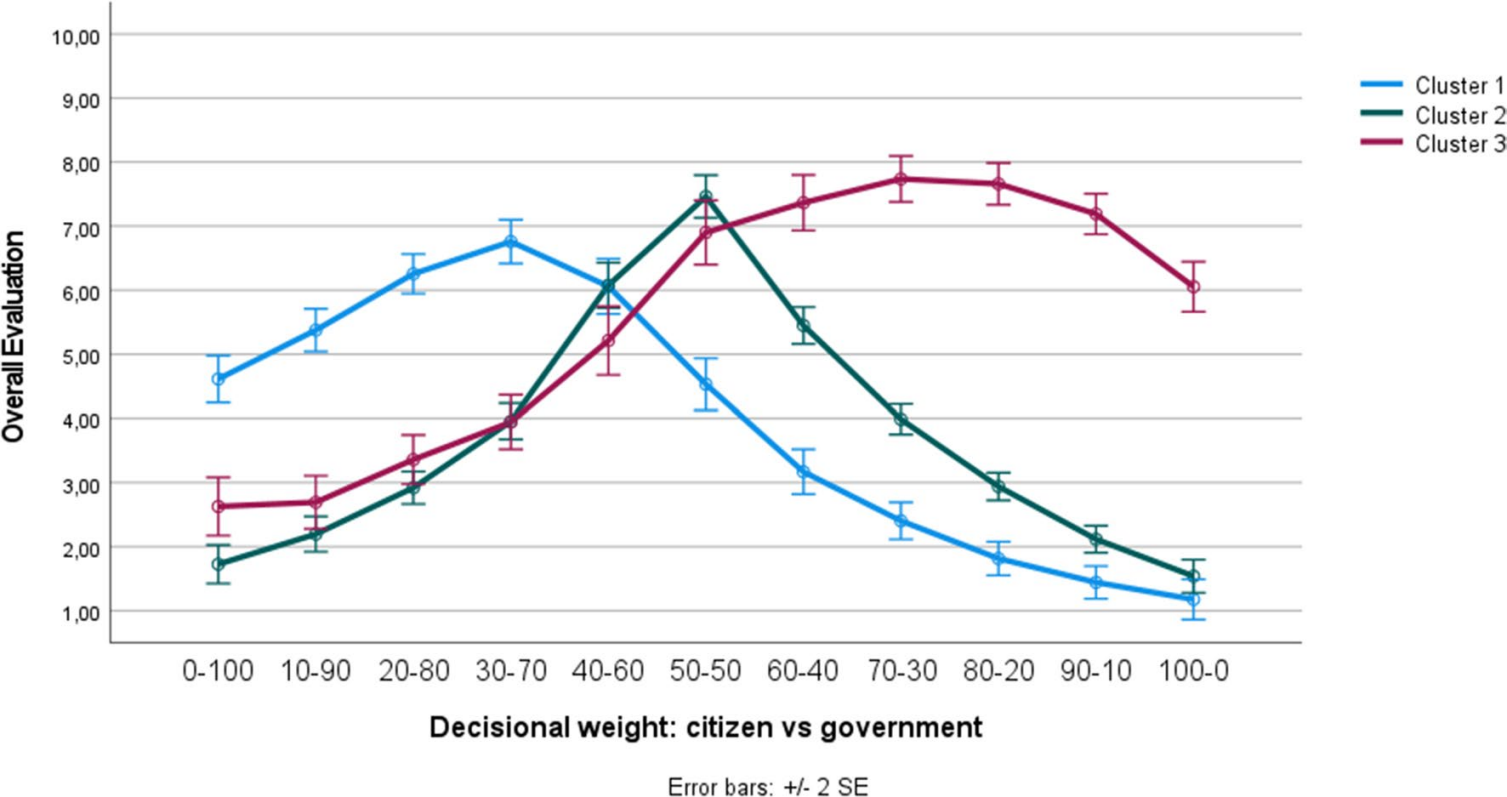
Three different reactions towards the decision-making models (Study 3).

It is important to realize, however, that the existence of such an overall pattern does not preclude the possibility of distinct clusters (i.e., subgroups) of individuals within the population, reacting differently to increasing levels of citizen (vs. government) involvement. To test this possibility, a *k*-means cluster analysis was conducted to categorize participants into different clusters, based on their responses to the different decision-making models. Because we hypothesized the existence of three subgroups within the citizenry (see “Introduction”), we extracted three distinct clusters, which are mutually exclusive (meaning that participants can only belong to one particular cluster). Figure 4 visualizes the corresponding curve of the three extracted clusters. Cluster 1 consists of a subgroup of participants (*n* = 94; 32.0%) who preferred the government to outweigh citizens. As shown in Fig. 4, in this first cluster, the decision-making model in which citizens have only 30% weight and the government has 70% weight was rated most positively. Cluster 2 contains a subgroup of participants (*n* = 139; 47.3%) who preferred the decision-making model in which citizens and the government both have an equal weight. Note that this particular subgroup closely mirrors the overall pattern (compare Cluster 2’s curve of Fig. 4 with the general pattern displayed in Fig. 3). Finally, Cluster 3 contains a subgroup of participants (*n* = 61; 20.7%) who preferred citizens to outweigh the government. Figure 4 illustrates that in this third cluster, the curve peaked when citizens have 70–80% weight and the government has only 20–30% weight. Interestingly, from Fig. 4 it can be derived that also in the ‘unequal balance’ clusters (i.e., Clusters 1 and 3), the model in which either the government or citizens have an absolute say (i.e., 100% weight) was clearly not considered the optimal model.

Finally, we conducted a multivariate analysis of variance (MANOVA), followed by post-hoc comparisons with Tukey HSD correction, to test if and how the three clusters (which were extracted based on participants’ responses to the different decision-making models) differ from each other with respect to our included demographics. The results of these analyses, which are summarized in Table 2, show that Cluster 1 consisted of significantly more male participants than Cluster 3 (*p* = 0.036)*.* Although Cluster 3 also appears to be the youngest and least educated, the three clusters did not differ significantly from each other in terms of participants’ age and education level (see Table 2).

**Table 2:** Means of the demographics as a function of the three clusters (Study 3).

Variable	Cluster 1	Cluster 2	Cluster 3
Age (in years)	39.44 (13.30)_a_	39.40 (14.57)_a_	37.44 (11.31)_a_
**Gender**	**0.60 (0.49)**_**a**_	**0.45 (0.50)**_**a,b**_	**0.39 (0.49)**_**b**_
Education	3.06 (0.79)_a_	2.82 (0.94)_a_	2.77 (0.69)_a_

Means with different subscripts in the same row differ significantly from one another (*p* < 0.05). The numbers in brackets are the standard deviations. Gender: 1 = male, 0 = other genders. Education: 1 = did not graduate, 2 = high school, 3 = bachelor’s degree, 4 = master’s degree, 5 = PhD or equivalent.

Significant values are in bold.

### Study 4

A consistent data pattern emerged in our previous studies, but in these studies we always used a rather broad (i.e., general) description of citizen participation, without a specific context. An important aim of this fourth study (*N* = 409) was to test if our previously obtained results also hold when participants are presented with more concrete (i.e., specific) cases. To this end, participants in the present study were asked to evaluate eleven decision-making models (the same ones as we used in Study 3) in the context of a specific local decision (see “Methods” for details on the included cases). The decision-making models were presented in a similar way as in our third study (i.e., through a within-subjects manipulation), but in the present study their presentation order was randomized. Moreover, the present study also aimed to explore if we could identify personal trait characteristics associated with the emerging subgroups. For that reason, we additionally also measured individual differences in terms of three ideological attitudes (RWA, LWA, and SDO), two types of cynicism (social and political), the six HEXACO personality dimensions, and ideological left–right self-placement.

#### Results of study 4

**Figure 5:**
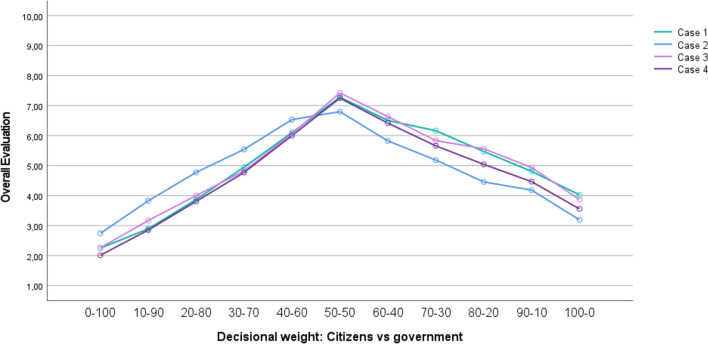
Overall evaluation (mean of appropriate, justified, and acceptable) in function of the decision-making models (Study 4). *Note.* Case 1 = repurposing of a vacant school building; Case 2 = reconstruction of a dangerous traffic situation; Case 3 = expansion of available sport facilities; Case 4 = location of a new shopping mall.

A repeated measures ANOVA revealed a significant effect of the decision-making models on participants’ overall evaluation (i.e., the mean of appropriate, justified, and acceptable), *F*(10, 396) = 128.17, *p* < 0.001, partial *η*^*2*^ = 0.764. Figure 5 displays the mean overall evaluation of the eleven decision-making models, separately for each of the four included cases. However, since the type of case did not interact significantly with the decision-making models, *F*(30, 1194) = 1.18, *p* = 0.231, partial *η*^*2*^ = 0.029, we decided to collapse the data across the four different local decisions in all subsequent analyses. Figure 5 illustrates that, for each of the four cases, participants’ overall evaluation of the decision-making models steadily increased up until the point where citizens and the local government both have a decisional weight of 50%. Once this overall optimum was reached, the curves started to drop. Similar to Study 3, pairwise comparisons with Sidak adjustment showed that the model in which citizens and the government both have a decisional weight of 50% again differed significantly from all the other models (all *p*s ≤ 0.001).

**Figure 6:**
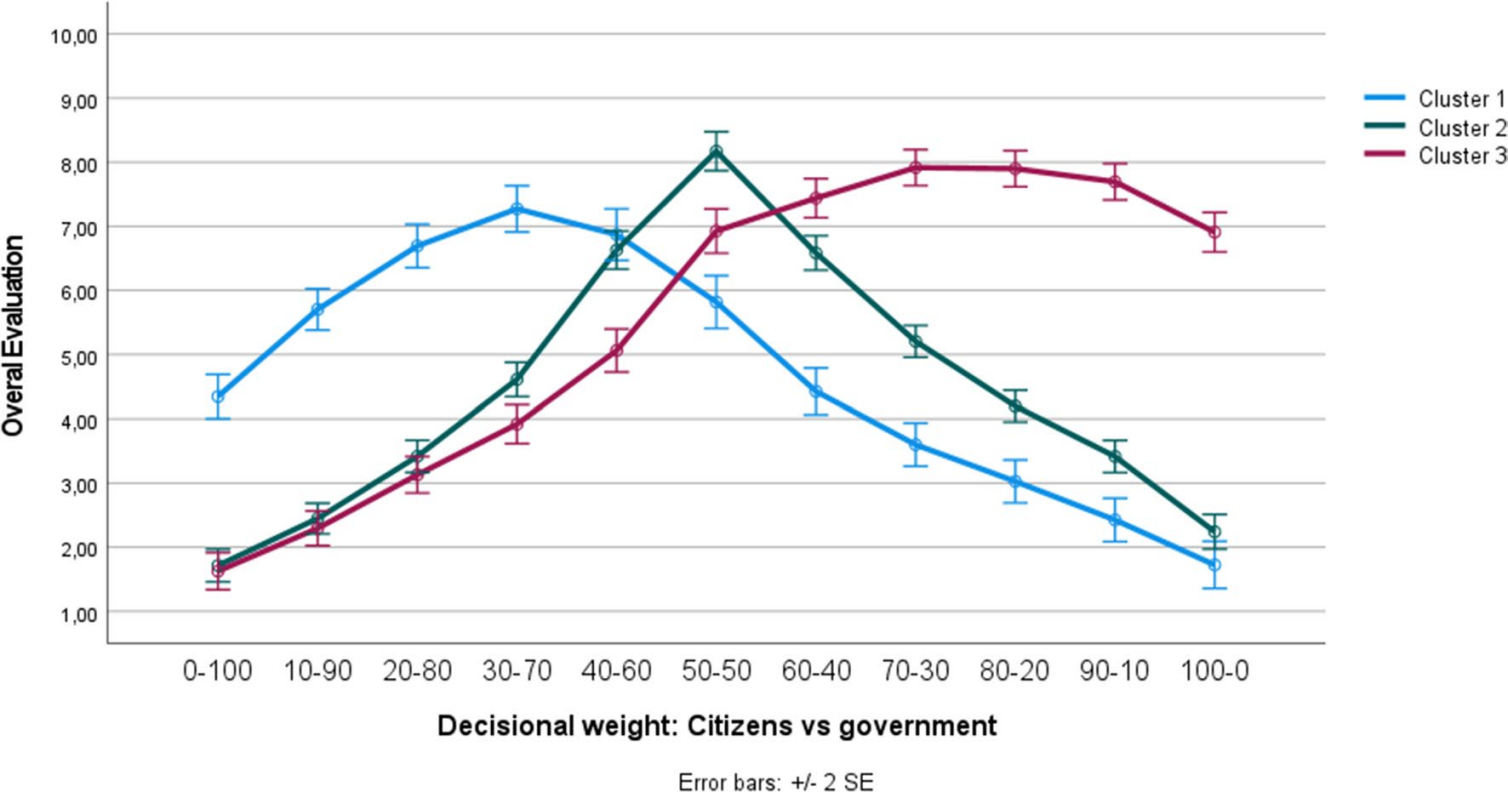
Three different reactions towards the decision-making models, collapsed across the four different cases (Study 4).

Preference patterns were once more investigated using a *k*-means cluster analysis, in which we again extracted three distinct and mutually exclusive clusters, based on how participants responded to the different decision-making models. Figure 6 visualizes the curve of the three extracted clusters. From this figure, it can be concluded that we again found a first subgroup of participants who preferred the government to have a greater input than citizens (Cluster 1; *n* = 96; 23.5%), a second subgroup who preferred citizens and the government to both have an equal input (Cluster 2;* n* = 176; 43.0%), and a third subgroup who preferred citizens to have a greater input than the government (Cluster 3; *n* = 137, 33.5%). Interestingly, the pattern of the three clusters extracted in the present study was virtually identical to the pattern of the three clusters extracted in Study 3 (we invite the reader to visually compare Fig. 4 with Fig. 6). And, as in Study 3, in none of the three clusters participants preferred a model in which either citizens or the government has complete say.

We then conducted a MANOVA, followed by post-hoc comparisons with Tukey HSD correction, to test if and how the three clusters (which were extracted based on participants’ responses to the different decision-making models) differ from one another in terms of our included demographics and individual trait measures. The results of these analyses are summarized in Table 3. As shown in this table, we found that participants in Cluster 3 turned out to be significantly lower educated than participants in Clusters 1 and 2 (both *p*s < 0.001). Moreover, Cluster 1 again consisted of more male participants and also appears to be the oldest, but the cluster differences in this regard were not statistically significant. In light of the individual trait measures, Table 3 shows that participants in Clusters 1 and 2 scored significantly lower on LWA than participants in Cluster 3 (both *p*s < 0.025). Additionally, participants in Cluster 1 also scored significantly lower on political cynicism (*p* = 0.034) and Emotionality (*p* = 0.014) than those in Cluster 3. However, for all the other individual trait measures under scrutiny, no significant differences between the three clusters were found (see Table 3).

**Table 3:** Means of the demographics and the individual trait measures as a function of the three emerging clusters (Study 4).

Variable	Cluster 1	Cluster 2	Cluster 3
Age (in years)	42.88 (11.90)_a_	41.31 (13.07)_a_	41.77 (14.67)_a_
Gender	0.53 (0.50)_a_	0.48 (0.50)_a_	0.48 (0.50)_a_
**Education**	**2.90 (0.81)**_**a**_	**2.81 (0.85)**_**a**_	**2.42 (0.76)**_**b**_
Right-wing authoritarianism	3.42 (1.14)_a_	3.63 (1.19)_a_	3.76 (1.22)_a_
**Left-wing authoritarianism**	**3.13 (0.89)**_**a**_	**3.18 (0.97)**_**a**_	**3.46 (0.92)**_**b**_
Social dominance orientation	2.77 (1.12)_a_	2.49 (1.09)_a_	2.45 (1.02)_a_
**Political cynicism**	**4.82 (1.14)**_**a**_	**5.07 (1.09)**_**a,b**_	**5.18 (0.99)**_**b**_
Social cynicism	4.51 (1.13)_a_	4.64 (1.04)_a_	4.71 (1.22)_a_
Honesty-humility	4.88 (0.91)_a_	4.84 (0.91)_a_	4.86 (0.94)_a_
**Emotionality**	**4.11 (1.09)**_**a**_	**4.27 (1.11)**_**a,b**_	**4.51 (0.97)**_**b**_
Extraversion	4.04 (1.00)_a_	4.11 (1.08)_a_	4.13 (1.07)_a_
Agreeableness	4.19 (0.97)_a_	4.45 (0.92)_a_	4.32 (0.92)_a_
Conscientiousness	4.97 (0.77)_a_	5.15 (0.85)_a_	4.94 (0.84)_a_
Openness to experience	4.76 (1.09)_a_	4.74 (1.03)_a_	4.59 (1.08)_a_
Left–right positioning	4.18 (2.02)_a_	4.34 (1.93)_a_	4.01 (2.03)_a_

Means with different subscripts in the same row differ significantly from one another (*p* < 0.05). The numbers in brackets are the standard deviations. Gender: 1 = male, 0 = other genders. Education: 1 = did not graduate, 2 = high school, 3 = bachelor’s degree, 4 = master’s degree, 5 = PhD or equivalent. The individual trait measures were all measured on seven-point Likert scales. Left–Right Positioning was measured on a scale from 0 (left) to 10 (right).

Significant values are in bold.

### Study 5

Our prior two studies revealed the same pattern of results for abstract (Study 3) and more concrete cases (Study 4). However, a limitation of the within-subjects designs that were used in these studies is that they required participants to judge the different decision-making models sequentially, that is, one at a time. Based on the evaluability framework^43,44^, it can be expected that people will be more sensitive to different degrees of citizen and government involvement when they evaluate the different decision-making models comparatively. Therefore, in this fifth and final study (*N* = 297) we used a pairwise comparison methodology to administer the different decision-making models. The same eleven models as in the prior two studies were included, which resulted in a total of 55 pairwise comparisons. For each of these comparisons, participants were forced to choose which of the two contrasted models they considered most appropriate (see “Methods” for more details). Two weeks later, during the second part of the study, participants (*N* = 240) completed the same individual trait measures as in Study 4.

#### Results of study 5

**Figure 7:**
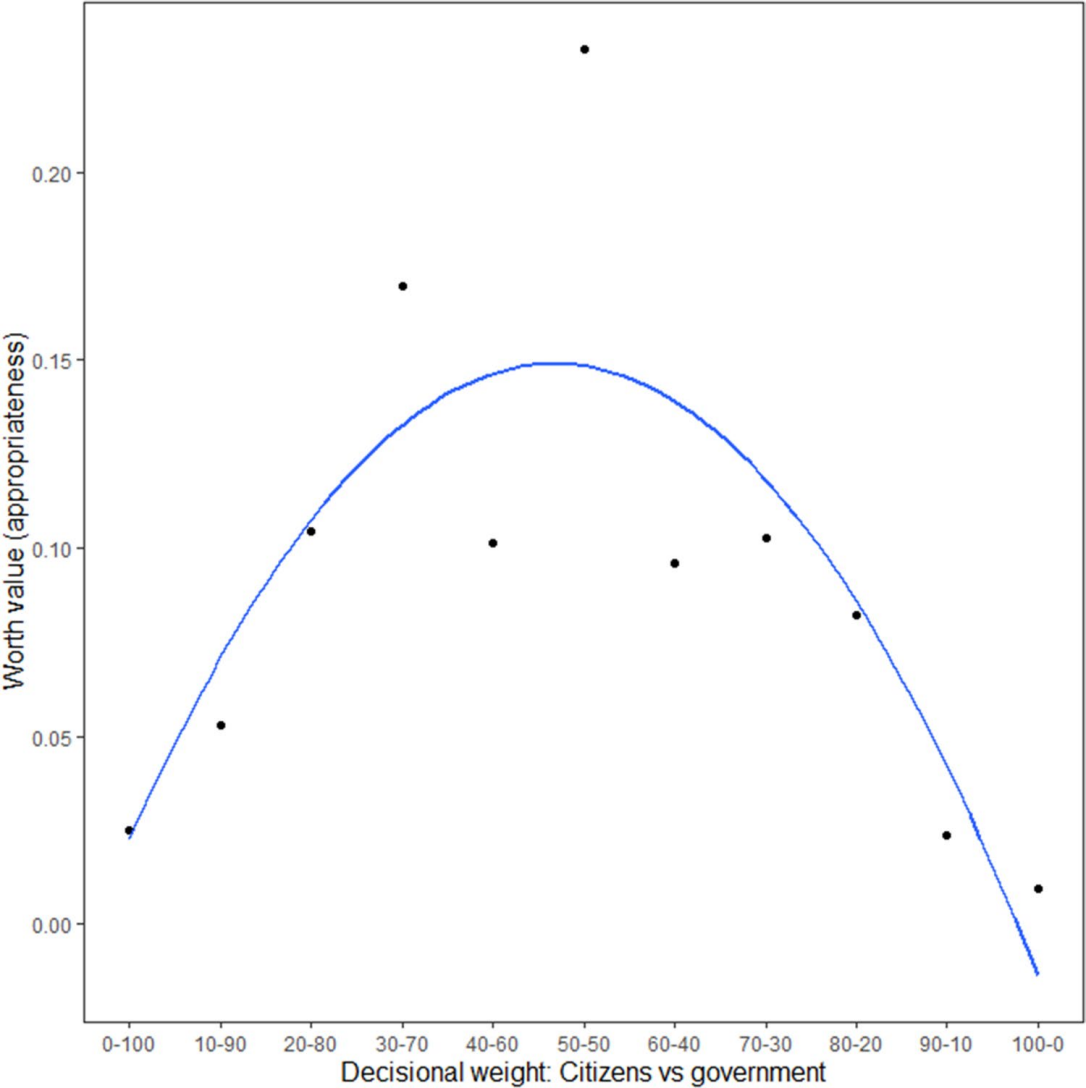
Appropriateness (estimated worth values) of the different decision-making models (Study 5). *Note.* Given two Models *A* and *B*, the probability that Model *A* is preferred over Model *B* is given by the worth of Model *A* divided by the sum of the worth of Models *A* and *B*. The dots reflect the estimated worth values. The trend line was added for interpretation purposes.

We first constructed a scale which numerically describes participants’ perceived appropriateness of the different decision-making models. This scale was estimated with a Bradley-Terry probability model using the Prefmod package^45^ in R (version 4.1.1). The location of each decision-making model on this scale was estimated by means of a worth value. These worth values quantify participants’ perceived appropriateness of a given decision-making model, relative to the other models. Figure 7 visualizes the estimated worth values of the different decision-making models. A visual inspection of this figure shows that the decision-making model that was perceived as most appropriate was again the one in which citizens and the government both have an equal weight.

**Figure 8:**
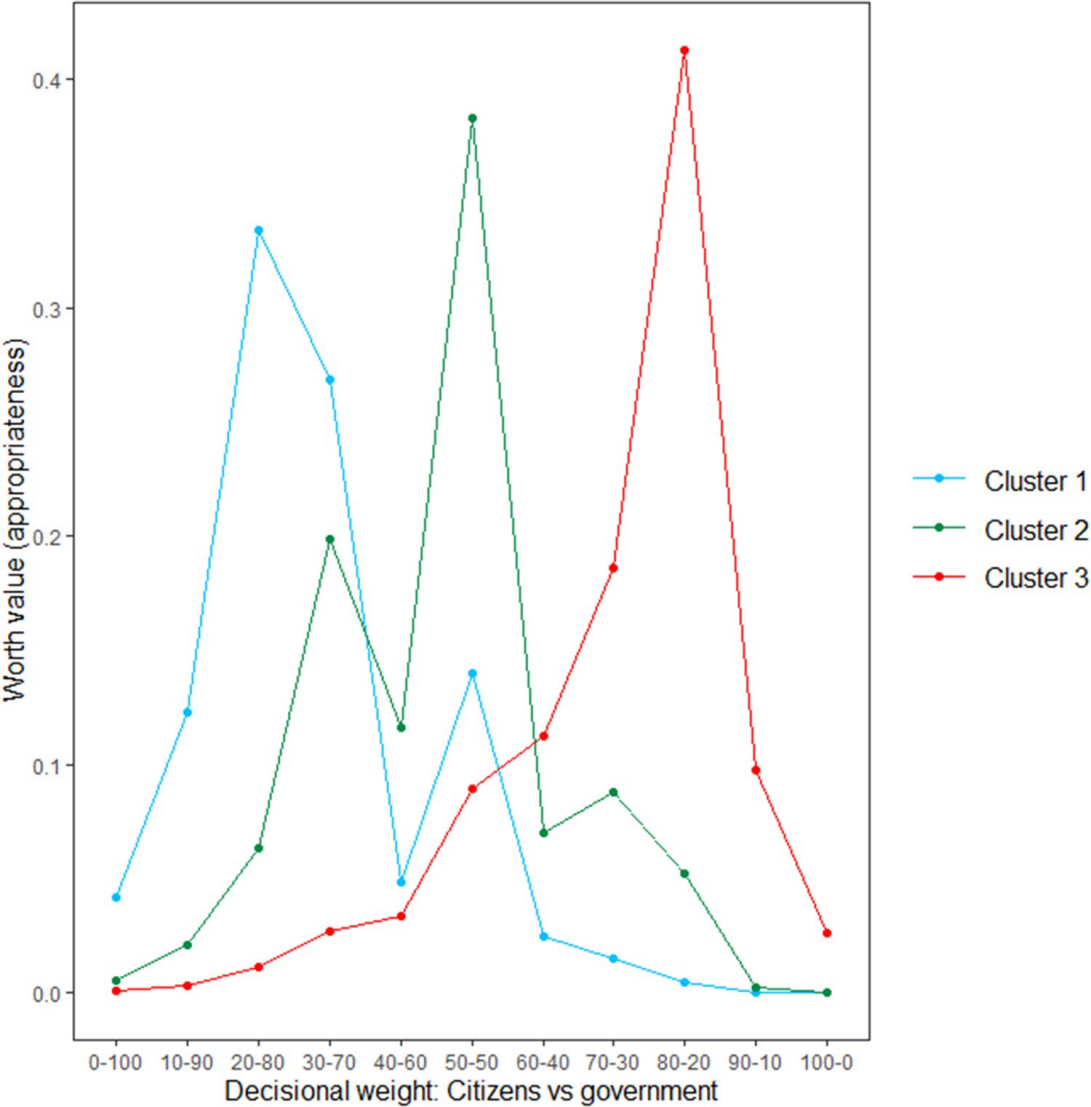
Three different reactions towards the pairwise comparisons (Study 5). *Note.* The dots represent the estimated worth values of the three clusters, the lines (which connect the dots) were added for visualization purposes.

Preference patterns were subsequently investigated using the klaR package^46^ in R, which allows for the clustering of categorical data. Based on participants’ responses to the pairwise comparisons, three distinct and mutually exclusive clusters were extracted, which are visualized in Fig. 8. This analysis revealed that the general pattern that we found again reflects the mere mean tendency of three distinct subgroups of citizens which each react differently to increasing levels of citizen involvement. As shown in Fig. 8, we again found a first subgroup who preferred the government to outweigh citizens (Cluster 1; *n* = 101; 34.0%), a second subgroup who preferred citizens and the government to both have an equal weight (Cluster 2; *n* = 116; 39.1%), and a third subgroup who preferred citizens to outweigh the government (Cluster 3; *n* = 80, 26.9%). Interestingly, although the estimated worth values displayed in Fig. 8 suggest that in none of the three clusters participants preferred a model in which either citizens or the government have a complete say, a closer inspection of participants’ responses nonetheless revealed that a small percentage of all people (about 10%) do seem to prefer a model in which either the government or citizens have full decisional control (i.e., 100% weight).

Finally, we again tested if and how the three clusters (which were extracted based on participants’ responses to the pairwise comparisons) differ from each other in terms of the included demographics and individual trait measures (which, as mentioned above, were measured during the second data collection phase). The results of these analyses, which are summarized in Table 4, clarify that participants in Cluster 1 were significantly older than those in Cluster 3 (*p* < 0.001). Similar to Study 3, Cluster 1 also consisted of significantly more male participants than Cluster 3 (*p* = 0.009). Although the three clusters did not differ significantly in terms of participants’ education level, the data again suggest that participants in Cluster 3 are the least educated. With regard to the individual trait measures, Table 4 shows that participants in Cluster 1 scored significantly lower on LWA than participants in Cluster 2 (*p* = 0.016) and Cluster 3 (*p* < 0.001). Similar to Study 4, we again found that participants in Cluster 1 also scored significantly lower on political cynicism than those in Cluster 3 (*p* < 0.001). Furthermore, in the presents study participants in Cluster 1 also scored significantly lower on Agreeableness than those in Cluster 2 (*p* = 0.045) and significantly higher on Conscientiousness than those in Cluster 3 (*p* = 0.006). For all the other traits, no significant differences between the three clusters were found (see Table 4).

**Table 4:** Means of the demographics and the individual trait measures as a function of the three emerging clusters (Study 5).

Variable	Cluster 1	Cluster 2	Cluster 3
**Age (in years)**	**43.49 (15.79)**_**a**_	**38.98 (14.11)**_**a,b**_	**35.45 (13.26)**_**b**_
**Gender**	**0.59 (0.49)**_**a**_	**0.49 (0.50)**_**a,b**_	**0.38 (0.49)**_**b**_
Education	2.76 (0.80)_a_	2.64 (0.84)_a_	2.57 (0.85)_a_
Right-wing authoritarianism	3.57 (1.18)_a_	3.63 (1.20)_a_	3.55 (1.27)_a_
**Left-wing authoritarianism**	**3.05 (0.89)**_**a**_	**3.42 (0.88)**_**b**_	**3.59 (0.93)**_**b**_
Social dominance orientation	2.68 (1.03)_a_	2.37 (1.01)_a_	2.52 (1.29)_a_
**Political cynicism**	**4.72 (1.07)**_**a**_	**5.02 (0.94)**_**a,b**_	**5.38 (1.05)**_**b**_
Social cynicism	4.60 (0.93)_a_	4.91 (0.95)_a_	4.96 (1.01)_a_
Honesty-humility	4.86 (0.95)_a_	4.96 (1.00)_a_	4.76 (0.92)_a_
Emotionality	4.19 (1.05)_a_	4.37 (0.93)_a_	4.38 (1.06)_a_
Extraversion	4.24 (0.94)_a_	4.09 (1.01)_a_	3.94 (1.11)_a_
**Agreeableness**	**4.10 (0.90)**_**a**_	**4.44 (1.00)**_**b**_	**4.16 (0.94)**_**a,b**_
**Conscientiousness**	**5.32 (0.84)**_**a**_	**5.03 (0.81)**_**a,b**_	**4.89 (0.82)**_**b**_
Openness to experience	4.73 (1.15)_a_	4.86 (0.90)_a_	4.70 (1.12)_a_
Left–right positioning	4.57 (2.01)_a_	3.84 (2.11)_a_	3.93 (2.27)_a_

Means with different subscripts in the same row differ significantly from one another (*p* < 0.05). The numbers in brackets are the standard deviations. Gender: 1 = male, 0 = other genders. Education: 1 = did not graduate, 2 = high school, 3 = bachelor’s degree, 4 = master’s degree, 5 = PhD or equivalent. The individual trait measures were all measured on seven-point Likert scales. Left–Right Positioning was measured on a scale from 0 (left) to 10 (right).

Significant values are in bold.

## Discussion

Over the last few decades, the nature of democracy has changed by allowing citizens to have a more direct say in policy decisions, especially at the local governmental level. An important question that has not been addressed yet, however, is how much direct involvement ordinary people think citizens should have relative to the government in local policy decision-making, and whether there are individual differences in this regard (and which). The present study aimed to shed light on these issues by using a so-called multi-study, multi-method approach. Below, we first elaborate on the theoretical and practical implications of our findings. Next, we describe the strengths and limitations of our studies, and formulate some recommendations for further research.

### Theoretical contributions

A first contribution of our work is that we consistently found that people do not want citizens (neither politicians) to have full decisional control in local policy-making decisions. This result is in line with prior research that reported a desire for so-called ‘hybrid’ models (see^7,23,47,48^), which combine elements of both representative and direct democracy. However, what the present study additionally contributes to literature is that we are able to identify a perceived ‘overall’ optimal level of citizen engagement. Across our five studies, we repeatedly found that, overall, the most preferred decision-making model is a balanced one in which citizens and the local government both have an equal decisional weight.

Importantly, however, our cluster analyses revealed that there is not just one perceived ‘optimal’ level of citizen involvement. The summarizing Table 5, which provides an overview of the cluster sizes across of different studies, clarifies that, on average, just over 30% of the citizenry prefers a decision-making model in which government involvement outweighs citizens’ involvement in policy decision-making (Cluster 1). For this first subgroup, the optimal model (i.e., apex of the preference curve) is one in which citizens have 30% and the government has 70% weight in policy decisions. Moreover, on average, nearly 40% of the citizenry mirrors the ‘overall’ pattern: They prefer a decision-making model in which citizens and the government are both equally involved (Cluster 2). Finally, on average, close to 30% of the citizenry prefers a decision-making model in which citizens have more weight than the government (Cluster 3). In this third subgroup, the optimal point of desired citizen decisional weight is 70–80% (vs. 20–30% government decisional weight).

**Table 5:** Summary of the cluster sizes across the different studies.

	Study 1	Study 3	Study 4	Study 5	Average
Cluster 1: Government outweigh citizens	37.0%	32.0%	23.5%	34.0%	31.6%
Cluster 2: Government and citizens equal weight	28.5%	47.3%	43.0%	39.1%	39.5%
Cluster 3: Citizens outweigh government	34.5%	20.7%	33.5%	26.9%	28.9%

The percentages indicate which proportion of the participants belonged to each of the three clusters. Participants in Study 1 were categorized based on their responses to the constant-sum question (i.e., those who preferred citizens to have less than 50% weight were assigned to Cluster 1, those who preferred citizens to have exactly 50% weight were assigned to Cluster 2, and those who preferred citizens to have more than 50% weight were assigned to Cluster 3). Participants in Studies 3, 4, and 5 were categorized based on their cluster membership. Given that in Study 2 the decision-making models were delivered using a between-subjects (instead of a within-subjects) manipulation, participants could not be categorized into different clusters. The average percentage (last column) reflects the mean of the different studies.

The above presented findings thus clarify that the preferred ‘overall’ pattern of equal involvement that we found in our studies actually reflects the mere mean tendency of three distinctive patterns. In this vein, it is particularly important to note that, in none of our studies, the cluster which prefers an equal weight balance between citizens and the government (Cluster 2) includes more than 50% of the participants (see Table 5 for details). So, although the subgroup that wants a balanced model is clearly the largest, the two other subgroups (who want more power for either the government or citizens) are together larger than those who prefer the balanced model. As such, one cannot straightforwardly conclude that a balanced model in which the government and citizens have a 50–50% involvement is supported by the majority. Yet, the overall patterns as well as the evaluation curves of the three subgroups suggest that the 50–50% balance is likely the most acceptable compromise for the three subgroups.

What the three identified subgroups do have in common, however, is that they all three clearly oppose a decision-making model in which either citizens or the local government has complete decision control. This finding challenges the assumption that citizen’ preferences can be reduced to a dichotomous position of decisional power lying *either* completely with the government (politicians and civil servants) *or* completely with citizens, as has been suggested in the literature (see^24,25^, for examples). Moreover, this findings also illustrates that democratic innovation in an attempt to reverse the decreased legitimacy of representative democracy is not a zero sum game between strengthening either representative, or direct democracy^49^. According to our study, most citizens prefer decision-making models that combine representative and direct approaches, thereby implicitly confirming the claim that democratic innovation through the introduction of participatory elements is also dependent on the involvement of politicians and on stable respected institutions^49^.

Our findings clarify that preferences about citizen involvement in decision-making also change according to several different demographical and individual trait characteristics. Specifically, we found that following characteristics and traits are overrepresented in the cluster that favors more citizen (compared to government) involvement: Female, younger, less educated, high left-wing authoritarianism, and high political cynicism. These patterns emerged over the different studies, although the differences were not always statistically significant (cf. results supra for more detail). These observations corroborate prior research showing that women exhibit higher support for participatory arrangements than men^47^, and showing that younger people are more in favor of direct democratic procedures than older citizens^12,15,24^. Also, earlier research found that less educated citizens^22,50^, citizens who distrust politicians^51^, and those who think that ‘things are not going well’^52^ are more likely to favor direct democracy. Moreover, our research adds to these findings by showing that people who score high on left-wing forms of authoritarianism also prefer citizens to have a greater say in policy decisions.

Together, our findings also shed a light on how people that are assumed to be at risk of an underprivileged position in society (such as women, youth, and lower educated individuals) look at democracy: They are clearly more in favour of more direct citizen involvement. Moreover, our results suggest that this position may be the result of a certain level of political cynicism, as well as left-wing opposition to traditional elites. Indeed, low trust in politics might be a trigger for wanting more direct citizen involvement in democracy. A possible explanation for this may be that these citizens feel that their interests are not taken care of enough by government and representative democracy. Another possible explanation may be that their actual political participation does not match with the desired political participation. The literature on the participation gap/paradox^53,54^ posits that highly educated, middle-aged men are overrepresented in all forms of political participation. Our results can then be interpreted as a desire for more participation opportunities by groups in society that are at higher risk of being (democratically) underprivileged.

A final observation of our research is that men and citizens above the average population age (who are often assumed to be overrepresented in participatory processes) actually seem most in favour of a relatively large role for the government. A first possible explanation for this seemingly counterintuitive finding might be that these citizens implicitly acknowledge that they are well-served by the key actors in traditional representative democracy. A plausible alternative explanation for this observation, however, stems from so-called ‘participatory frustration’^55^, which refers to the frustration that might occur after engaging in dissatisfying participatory processes. According to this perceptive, those who have been most involved in participatory initiatives (like men and older citizens) might also be most disillusioned by inflated expectations and the lack of impact upon public policy. Future research is needed to verify these assumptions.

### Practical implications

Our research also has some practical implications for policy-makers at the local governmental level, who are tasked with developing effective citizen participation processes. A first takeaway point for practitioners is that, although citizens clearly want to be involved in local policy decisions, this does not imply that they want to maximally exclude governmental actors from such decisions. Indeed, our results suggest that the role of traditional representative democratic actors is still valued by the vast majority of the citizenry, albeit with varying degrees, dependent on individual traits, which complicates the search for the right balance between governmental and citizen decisional weight in policy decision-making. Our findings illustrate that anything between 30 and 70% citizen weight will work for most citizens, with 50% weight for citizens being the ‘overall’ perceived optimum.

Despite this preferred ‘overall’ pattern of equal involvement, we identified three subgroups within the citizenry, who each display a different preference curve. Particularly interesting in this regard is that we found that the subgroup who wants more direct citizen decisional weight is characterized by higher levels of political cynicism and left-wing authoritarianism than the other two subgroups. Although these citizens seem to be dissatisfied with how representative democracy is currently organized, they do not want to exclude the government entirely from policy decisions. Offering these citizens more participation opportunities might be a feasible way to address their discontent and enhance their legitimacy perceptions of the system, through cooperation with the traditional political and administrative decision-makers in a more hybrid form of democracy.

### Strengths, limitations, and future research

We employed a multi-study, multi-method approach to empirically investigate how much weight ordinary people prefer citizens and the government to have, relative to each other, in local policy decisions. To our knowledge, no prior studies have undertaken such an endeavor. The most important strength of the present set of studies is that we used a variety of different research methods and designs (see Table 1 for an overview). That is, we first conducted an explorative study in which we employed a constant-sum approach to unravel the preferred relative amount of citizen and government involvement (Study 1). This explorative study was followed by a series of studies in which the different weight distributions were administered to participants using a between-subject design (Study 2), within-subjects designs (Studies 3 and 4), and pairwise comparisons (Study 5). Moreover, in our studies we included both general (Studies 1, 2, 3, and 5) and specific cases (Study 4). The fact that we could replicate our main findings using this variety in methods and designs bolsters confidence in the robustness and generalizability of the reported findings. However, because not all of our studies used a strict experimental design, caution should be exerted when drawing causal inferences from the present set of studies.

Of course, the present study is not without limitations. The most critical limitation of our research is that we asked participants to evaluate abstract conceptualizations of ‘decisional power balance’, that is, the relative amount of decisional weight that citizens and the government would have in local decisions. Because of this, the ideal level of ‘overall’ citizen involvement that we found in our studies should be seen as more symbolical. Although we found that, overall, people prefer a balanced model in which citizens and the government both have an equal decisional weight, due to our abstract approach, it remains unclear how such a 50-50% involvement could actually translate into concrete operationalizations in policy-making. Such a balance might, for example, translate into an arrangement wherein half of the issues are decided by the government and the other half by the citizenry, or rather that the recommendations (or direct votes) of citizens and the government on a specific issue are weighed equally, or that citizens and government each have decision power on how to allocate half of the available resources. Future research in this domain is therefore encouraged to investigate how the different power balances that we examined in our study can best be translated into practice.

Another limitation of our study is that we did not provide any information on the number of citizens that would be invited to participate (e.g., whole population or selected sample), the selection of participating citizens (e.g., self-selection or random selection), and the way in which participating citizens would be involved (e.g., vote-centric or talk-centric way). Relatedly, we also did not collect data on participants’ prior experiences with participatory arrangements at the local governmental level. Previous experience with, or involvement in participatory initiatives may nonetheless influence people’s preferences and wishes. Therefore, we strongly encourage future research in this domain to also take such variables into consideration, and investigate their potential impact.

Finally, even though we repeatedly found three clusters of people who react differently to increasing levels of citizen involvement, we did not find many personal trait differences between these clusters. A possible explanation for this observation might reside in the fact that each cluster indeed comprises individuals with a shared preference vis-a-vis participation, but possibly for different reasons. For example, it is feasible that there are at least two very different reasons why people in Cluster 3 (i.e., the cluster which prefers citizens to outweigh the government) endorse greater citizen involvement in policy decisions. That is, striving for more citizen involvement has been attributed to dissatisfied citizens who look for an alternative for representation as well as to politically engaged citizens who want more opportunities to participate in politics (see^11,15,51^). As such, it is possible that the subgroup of citizens who prefer citizens to outweigh the government actually consists of both ‘enraged’ (dissatisfied) and ‘engaged’ citizens. Given that such enraged and engaged citizens might be characterized by rather different personality traits, this mixed underlying profile within the same ‘preference cluster’ may hamper the emergence of clear trait differences between the different clusters. Further research is also needed to examine the variety of motives that may underlie each cluster.

## Methods

Ethical approval for the present studies was obtained from the Ethics Committee of the Faculty of Economics and Business Administration at Ghent University (Ref: UG-EB 2022-A), where the first author and last author are affiliated. All studies were performed in accordance with the 1964 Helsinki Declaration and its later amendments. Informed consent was obtained from every participant.

### Study 1

The aim of our first study was to explore the preferred amount of citizen involvement in local policy-making. To this end, we recruited a sample of 200 UK participants through Prolific (100 men, *M*_*age*_ = 39.11, *SD* = 13.64; see Table B.1 of Appendix B for more details). At the start of this study, participants were presented with the following introductory statement:

Citizen participation refers to direct involvement of the public in policy-making by the (local) government. In recent years, governments have increasingly allowed citizens to participate in local policy decisions (such as the repurposing of vacant buildings, the reconstruction of dangerous traffic situations, the expansion of available sport facilities, etc.). Importantly, citizens and their local government can have different weights in the policy-making process.

We subsequently employed a constant-sum scale to assess the relative amount of say that participants prefer citizens and their local government to have. To this end, participants were asked how much decisional weight they prefer local citizens to have and how much decisional weight they prefer the local government to have in the policy-making process. For both local citizens and the local government, participants were asked to fill in a number between 0 and 100 (with 0 indicating no say at all and 100 indicating absolute say); the sum of these two numbers had to equal 100.

### Study 2

#### Sample and design

Our second study aimed to investigate how people evaluate different degrees of citizen and government involvement in the context of a local policy-making scenario. We recruited an initial sample of 275 UK participants through Prolific. Five participants (1.8%) were excluded from the analyses because they failed our manipulation check (see below for details). The remaining 270 participants (135 men) were on average 37.89 years old (*SD* = 13.87; see Table B.1 of Appendix B for more details). The independent variable consisted of five different decision-making models (ranging from no citizen involvement up to full citizen control), which were manipulated using a between-subjects design.

#### Procedure

At the beginning of the study, participants were presented with the same introductory statement as in Study 1. After reading this statement, participants were randomly assigned to one of five experimental conditions that varied the decisional weight that local citizens and the local government have in the policy-making scenario. In the first condition (*n* = 54), citizens have 0% and the government has 100% weight. In the second condition (*n* = 54), citizens have 25% and the government has 75% weight. In the third condition (*n* = 53), citizens and the government both have 50% weight. In the fourth condition (*n* = 54), citizens have 75% and the government has 25% weight. In the fifth condition (*n* = 55), citizens have 100% and the government has 0% weight. Figure C.1 of Appendix C illustrates how this information was communicated to the participants.

#### Measures

As a manipulation check, we first asked participants to indicate how much decisional weight citizens and the government had in the presented scenario. Five participants provided answers that were inconsistent with their allocated condition, and were therefore removed from the analyses. In each condition, we subsequently asked participants: “To what extent do you find this decision-making model: acceptable, legitimate, fair, democratic, effective, efficient, appropriate, and justified.” Participants could answer each of these questions on a ten-point Likert scale, ranging from (1) *not at all* to (10) *very much so*. Because responses on these eight items all loaded on one single factor (which explained 76.6% of the variance; see Table D.1 of Appendix D for the factor loadings), they were averaged into a general scale measure which constitutes participants’ overall evaluation of the extent to which the presented decision-making model reflects ‘good governance’ (*M* = 5.05, *SD* = 2.38, Cronbach’s alpha = 0.96).

### Study 3

#### Sample and design

A sample of 300 UK participants was recruited through Prolific. Six of them (2.0%) were excluded from the analyses for failing our check questions (see below). The remaining 294 participants (142 men) were on average 39.01 years old (*SD* = 13.53; see Table B.1 of Appendix B for more details). The independent variable consisted of eleven different decision-making models, which were presented to participants using a within-subjects manipulation.

#### Procedure

Participants were asked to read the same introductory statement as in the previous two studies. Afterwards, they were presented with the different decision-making models. In the present study, we included a total of eleven different decision-making models, ranging from no citizen involvement up to full citizen control (citizen vs. government weight: 0% vs. 100%, 10% vs. 90%, 20% vs. 80%, 30% vs. 70%, 40% vs. 60%, 50% vs. 50%, 60% vs. 40%, 70% vs. 30%, 80% vs. 20%, 90% vs. 10%, 100% vs. 0%). These decision-making models were presented to participants in a fixed order, starting with the model in which citizens have 0% weight and ending with the model in which citizens have 100% weight.

#### Measures

For each decision-making model, we first asked participants, as a manipulation check, to indicate the amount of decisional weight that citizens and the government have according to that particular model. The six participants who provided incorrect answers to more than one of these check questions were excluded from the analyses. We then asked participants—for each of the eleven decision-making models—to what extent they deemed that particular decision-making model appropriate, justified, and acceptable (1 = *not at all*, 10 = *very much so*). These three items were selected because they showed the highest factor loadings in Study 2 (see Table D.1 of Appendix D). For each decision-making model, participants’ scores on these three items were averaged into a general scale reflecting participants’ overall positive evaluation (Cronbach’s alpha = [0.97, 0.98]).

### Study 4

#### Sample and design

A total of 426 participants took part in our fourth study. As a result of failing the included check questions (see below for details), 17 participants (4.0%) were removed from the analyses. The remaining 409 participants (202 males) were on average 41.83 years old (*SD* = 13.36; see Table B.1 of Appendix B for more detailed descriptive statistics). We employed a mixed-factorial design in which we included eleven decision-making models (the same constellations as we used in Study 3) as the within-subjects factor and four different cases (corresponding to four different local decisions) as the between-subjects factor.

#### Procedure

Participants were presented a similar introductory statement as in the previous studies. However, to verify the possibility that people’s assessment of citizen (vs. government) involvement may depend on the specific decision that has to be made, we manipulated to which particular decision the decision-making models applied. More precisely, participants were asked to evaluate the decision-making models for one of the following four local decisions: The repurposing of a vacant school building (Case 1; *n* = 112); the reconstruction of a dangerous traffic situation (Case 2; *n* = 91); the expansion of available sport facilities (Case 3; *n* = 105); or the location of a new shopping mall (Case 4;* n* = 101). More detailed information about the formulation of these four different cases can be found in Appendix E (Figs. E.1–E.4). In each of these four between-subjects conditions, the eleven decision-making models (which again ranged from 0% citizen and 100% government weight up to 100% citizen and 0% government weight, in small steps of 10%) were presented in a similar way as in Study 3 (i.e., through a within-subjects manipulation). Importantly, in the present study the order in which these decision-making models were presented to participants was randomized.

#### Measures

For each decision-making model, we first asked participants, as a manipulation check, to indicate the weight that citizens and the government have according to that particular model. Seven participants who provided incorrect answers to more than one of these check questions were excluded from the analyses. Similar to Study 3, we then asked participants—for each of the eleven decision-making models—to what extent they found that particular model appropriate, justified, and acceptable (1 = *not at all*, 10 = *very much so*). Again, for each model these three items were averaged into a general scale measure (Cronbach’s alpha = [0.98, 0.99]; collapsed across the four cases).

After participants had rated the different decision-making models, we tested if they were able to recall to which particular decision the different models applied, and provided the four possible cases as response options. An additional eight participants’ responses were inconsistent with their allocated condition, and were therefore also removed from the analyses. Moreover, as an additional control, we also included two attention checks in the individual trait measures (e.g., “please select the first response option”), which led to the further exclusion of two extra participants.

#### Individual traits

At the end of the study, participants were presented with the individual trait measures. With exception of the ideological left–right self-placement scale, these measures were all rated on seven-point Likert scales ranging from (1) *strongly disagree* to (7) *strongly agree*. Negatively phrased items were reversed before the scale scores were constructed. The means, standard deviations, Cronbach’s alphas, and intercorrelations among the individual trait measures can be found in Table F.1 of Appendix F. The full item lists are included in Appendix G.

*Ideological attitudes* Ideological attitudes were assessed by means of a ten-item version of the Right-Wing Authoritarianism (RWA) scale^26^, an 18-item version of the Left-Wing Authoritarianism (LWA) scale^30^, and a 14-item version of the Social Dominance Orientation (SDO) scale^31^. Sample items are: “Being kind to loafers or criminals will only encourage them to take advantage of our weakness, so it is best to use a firm, tough hand when dealing with them” (RWA), “The rich should be stripped of their belongings and status” (LWA), and “To get ahead in life, it is sometimes necessary to step on others” (SDO).

*Political and social cynicism* To measure these two different types of cynicism, we employed the eight-item political cynicism scale as well as the six-item social cynicism scale of Pattyn et al.^32^. Sample items are: “People are very frequently manipulated by politicians” (political cynicism) and “I am often sceptical and cynical about people’s intentions” (social cynicism).

*HEXACO personality dimensions* The six personality dimensions of Honesty-Humility, Emotionality, Extraversion, Agreeableness, Conscientiousness, and Openness to Experience were measured using the HEXACO-60 personality inventory^33^. Each of these six dimensions was measured with a set of ten items. Sample items are: “I wouldn’t use flattery to get a raise or promotion at work, even if I thought it would succeed” (Honesty-Humility), “I sometimes can’t help worrying about little things” (Emotionality), “In social situations, I’m usually the one who makes the first move” (Extraversion), “I rarely hold a grudge, even against people who have badly wronged me” (Agreeableness), “I plan ahead and organize things, to avoid scrambling at the last minute” (Conscientiousness), and “I would enjoy creating a work of art, such as a novel, a song, or a painting” (Openness to Experience).

*Left–right positioning* Finally, participants’ self-placement on an ideological left–right scale was measured using a single item. Participants were first told that ‘left’ and ‘right’ are concepts often used to describe political attitudes. They were then asked to indicate their own position, using a scale ranging from 0 (*left*) to 10 (*right*).

### Study 5

Study 5 consisted of two different data collection phases. During the first phase, we initially recruited 300 participants and measured their reactions towards the different decision-making models as well as the demographical variables. We excluded three participants (1.0%) who failed our comprehension check (see below). The remaining 297 participants (147 males) were on average 39.56 years old (*SD* = 14.78). During the second phase—which took place about two weeks after the first phase—the 297 participants who correctly answered our comprehension check during the first data collection phase were contacted again with the question to complete the individual trait measures. Of the 297 contacted participants, 247 (83.2%) participated in the second phase of our study. Seven of them (2.8%) were excluded because they failed at least one of the two included attention checks in the second phase (e.g., “please select the first response option”). Of the remaining 240 participants (i.e., 80% of the original sample), there were 119 male participants (*M*_age_ = 41.62, *SD* = 15.02). More detailed descriptive statistics can be found in Table B.1 of Appendix B.

#### Phase 1: pairwise comparisons

During the first data collection phase, participants were presented the same introductory statement (reflecting the abstract case) as in Studies 1, 2 and 3. Like in Studies 3 and 4, the included decision-making models again ranged from 0% citizen and 100% government weight up to 100% citizen and 0% government weight, in increments of 10%. However, in this study the resulting eleven decision-making models were presented to participants in pairs—each pair contrasted two decision-making models. This method eventually resulted in a total of 55 pairwise comparisons for each participant to complete. Appendix H (Table H.1) provides an overview of the included comparisons.

We first provided participants with an example of such a pairwise comparison, and asked them, as a comprehension check, how much decisional weight citizens and the government had in the two contrasted decision-making models. The three participants who failed to answers these questions correctly were excluded from the analyses. Thereafter, participants were presented with the 55 pairwise comparisons (see Fig. I.1 of Appendix I for an example of how these comparisons were presented to participants). For each of these 55 pairwise comparisons, participants were forced to choose which of the two contrasted decision-making models (*Model A* or *Model B*) they considered most appropriate. In order to avoid potential sequence effects, the presentation order of these comparisons was randomized.

#### Phase 2: individual traits

During the second data collection phase, the three ideological attitudes (RWA, LWA, and SDO), the two cynicism types (political and social cynicism), the six HEXACO personality dimensions (Honesty-Humility, Emotionality, Extraversion, Agreeableness, Conscientiousness, and Openness to Experience), and our ideological left–right self-placement scale were measured using the same items as in Study 4. The means, standard deviations, Cronbach’s alphas, and intercorrelations among these individual trait measures are presented in Table J.1 of Appendix J.

### Ethical approval

Ethical approval for the present research was obtained from the Ethics Committee of the Faculty of Economics and Business Administration at Ghent University (Ref: UG-EB 2022-A). All studies were performed in accordance with the 1964 Helsinki Declaration and its later amendments. Informed consent was obtained from every participants.

## Supplementary Information

Supplementary Information.

## Data Availability

The data that are reported in the present manuscript are made publicly available and can be openly accessed through Open Science Framework: https://osf.io/zs9cj/. The data and data analysis scripts can also be requested from the first author: tessa.haesevoets@ugent.be.
