# Supplementary material for: The public’s preferred level of involvement in local policy-making
Source: Sci Rep. 2023 May 2;13:7146. doi: 10.1038/s41598-023-34282-w (PMC10154419; doi:10.1038/s41598-023-34282-w)
Supplement: Supplementary file 1 — Supplementary Information. [file 41598_2023_34282_MOESM1_ESM.docx]

**APPENDIX A**

Results of the power sensitivity analyses

After data collection, with exception of Study 1 (which was explorative in nature), for each study we ran a power sensitivity analysis to assess whether our sample size was adequate for uncovering the reported effects. The results of these sensitivity analyses illustrate that our studies were all sufficiently powered to detect the reported effects:

- Study 2 (*N* = 270) had 80% power to detect main effects of size (Cohen’s) *f* = 0.09 (as a point of reference, observed *f* in this study = 0.80).
- Study 3 (*N* = 294) had 80% power to detect main effects of size *f* = 0.24 (observed *f* in this study = 1.19).
- Study 4 (*N* = 409) had 80% power to detect main effects of size *f* = 0.20 (observed *f* in this study = 0.92).
- Study 5 (*N* = 297) had 80% power to detect main effects of size *f* = 0.32 (observed *f* in this study = 0.70).

**APPENDIX B**

**TABLE B.1.** Detailed descriptive statistics (gender and education) of the different samples.

| Gender category | | Frequency (%) | Education level | Frequency (%) |
| --- | --- | --- | --- | --- |
| **Study 1 (*N* = 200)** | | | | |
| Male | | 100 (50.0%) | Did not graduate | 7 (3.5%) |
| Female | | 99 (49.5%) | High school | 87 (43.5%) |
| Non-binary / third gender | | 0 (0.0%) | Bachelor’s degree | 77 (38.5%) |
| Prefer not to say | | 1 (0.5%) | Master’s degree | 23 (11.5%) |
|  | |  | PhD or equivalent | 6 (3.0%) |
| **Study 2 (*N* = 270)** | | | | |
| Male | | 135 (50.0%) | Did not graduate | 11 (4.1%) |
| Female | | 130 (48.1%) | High school | 91 (33.7%) |
| Non-binary / third gender | | 4 (1.5%) | Bachelor’s degree | 123 (45.6%) |
| Prefer not to say | | 1 (0.4%) | Master’s degree | 39 (14.4%) |
|  | |  | PhD or equivalent | 6 (2.2%) |
| **Study 3 (*N* = 294)** | | | | |
| Male | | 142 (48.3%) | Did not graduate | 9 (3.1%) |
| Female | | 148 (50.3%) | High school | 88 (29.9%) |
| Non-binary / third gender | | 1 (0.3%) | Bachelor’s degree | 134 (45.6%) |
| Prefer not to say | | 3 (1.0%) | Master’s degree | 53 (18.0%) |
|  | |  | PhD or equivalent | 10 (3.4%) |
| **Study 4 (*N* = 409)** | | | | |
| Male | 202 (49.4%) | | Did not graduate | 20 (4.9%) |
| Female | 204 (49.9%) | | High school | 156 (38.1%) |
| Non-binary / third gender | 1 (0.2%) | | Bachelor’s degree | 169 (41.3%) |
| Prefer not to say | 2 (0.5%) | | Master’s degree | 56 (13.7%) |
|  |  | | PhD or equivalent | 8 (2.0%) |
| **Study 5; Phase 1 (*N* = 297)** | | | | |
| Male | 147 (49.5%) | | Did not graduate | 14 (4.7%) |
| Female | 148 (49.8%) | | High school | 122 (41.1%) |
| Non-binary / third gender | 2 (0.7%) | | Bachelor’s degree | 116 (39.1%) |
| Prefer not to say | 0 (0.0%) | | Master’s degree | 40 (13.5%) |
|  |  | | PhD or equivalent | 5 (1.7%) |

*Table B.1 continued*

| Gender category | | Frequency (%) | Education level | Frequency (%) |
| --- | --- | --- | --- | --- |
| **Study 5; Phase 2 (*N* = 240)** | | | | |
| Male | 119 (49.6%) | | Did not graduate | 14 (5.8%) |
| Female | 119 (49.6%) | | High school | 93 (38.8%) |
| Non-binary / third gender | 2 (0.8%) | | Bachelor’s degree | 94 (39.2%) |
| Prefer not to say | 0 (0.0%) | | Master’s degree | 34 (14.2%) |
|  |  | | PhD or equivalent | 5 (2.1%) |

**APPENDIX C**

| **CITIZENS** | **GOVERNMENT** |
| --- | --- |
| Local citizens have a  decisional weight of 0% | The local government has a  decisional weight of 100% |
| => This specific decision-making model implies that local citizens have no say, while the local government has complete say in policy-making. | |

**FIGURE C.1.** Example of the presentation of a decision-making model (Study 2).

**APPENDIX D**

**TABLE D.1.** Factor loadings (Study 2).

| Item | Factor loading |
| --- | --- |
| Appropriate | .95 |
| Justified | .95 |
| Acceptable | .93 |
| Fair | .92 |
| Effective | .90 |
| Democratic | .80 |
| Legitimate | .80 |
| Efficient | .73 |

**APPENDIX E**

Citizen participation refers to direct involvement of the public in policy-making by the (local) government. In recent years, governments have increasingly allowed citizens to participate in local policy decisions.

An example of such a local policy decision concerns the repurposing of a vacant building. Imagine, for instance, that in your town the former high school has been vacant for years, and that this building needs to be given a new purpose. Importantly, local citizens and the local government can have different weights in deciding what should happen with the vacant school building.

On the next pages, we will present you with several different decision-making models (a decisional weight of 0% indicates no say at all, while a decisional weight of 100% indicates complete say).

**FIGURE E.1.** Information provided to participants in Case 1 (Study 4).

Citizen participation refers to direct involvement of the public in policy-making by the (local) government. In recent years, governments have increasingly allowed citizens to participate in local policy decisions.

An example of such a local policy decision concerns the reconstruction of a dangerous traffic situation. Imagine, for instance, that in your town there is an intersection that has caused several accidents, and which has to be redesigned. Importantly, local citizens and the local government can have different weights in deciding how this dangerous intersection should be reconstructed.

On the next pages, we will present you with several different decision-making models (a decisional weight of 0% indicates no say at all, while a decisional weight of 100% indicates complete say).

**FIGURE E.2.** Information provided to participants in Case 2 (Study 4).

Citizen participation refers to direct involvement of the public in policy-making by the (local) government. In recent years, governments have increasingly allowed citizens to participate in local policy decisions.

An example of such a local policy decision concerns the expansion of the available sport offerings. Imagine, for instance, that there are insufficient sport facilities in your town, and that the range of sports on offer should be expanded. Importantly, local citizens and the local government can have different weights in deciding how to expand the current sport offerings.

On the next pages, we will present you with several different decision-making models (a decisional weight of 0% indicates no say at all, while a decisional weight of 100% indicates complete say).

**FIGURE E.3.** Information provided to participants in Case 3 (Study 4).

Citizen participation refers to direct involvement of the public in policy-making by the (local) government. In recent years, governments have increasingly allowed citizens to participate in local policy decisions.

An example of such a local policy decision concerns the location of a new shopping mall. Imagine, for instance, that a new shopping mall will be built in your town, but the exact location of this shopping mall still has to be decided. Importantly, local citizens and the local government can have different weights in deciding where to build this new shopping mall.

On the next pages, we will present you with several different decision-making models (a decisional weight of 0% indicates no say at all, while a decisional weight of 100% indicates complete say).

**FIGURE E.4.** Information provided to participants in Case 4 (Study 4).

**APPENDIX F**

**TABLE F.1.** The means, standard deviations, Cronbach’s alphas, and intercorrelations among the individual trait measures (Study 4).

| Variable | *M* | *SD* | α | 1. | 2. | 3. | 4. | 5. | 6. | 7. | 8. | 9. | 10. | 11. |
| --- | --- | --- | --- | --- | --- | --- | --- | --- | --- | --- | --- | --- | --- | --- |
| 1. Right-Wing Authoritarianism | 3.62 | 1.19 | .88 | -- |  |  |  |  |  |  |  |  |  |  |
| 2. Left-Wing Authoritarianism | 3.26 | 0.94 | .87 | -.11* | -- |  |  |  |  |  |  |  |  |  |
| 3. Social Dominance Orientation | 2.54 | 1.08 | .92 | .52** | -.27** | -- |  |  |  |  |  |  |  |  |
| 4. Political Cynicism | 5.05 | 1.08 | .87 | .09 | .21** | -.00 | -- |  |  |  |  |  |  |  |
| 5. Social Cynicism | 4.63 | 1.12 | .89 | .33** | .15** | .18** | .44** | -- |  |  |  |  |  |  |
| 6. Honesty-Humility | 4.86 | 0.92 | .71 | -.14** | -.05 | -.28** | -.19** | -.26** | -- |  |  |  |  |  |
| 7. Emotionality | 4.32 | 1.07 | .83 | -.09 | .24** | -.22** | .04 | 0.06 | .06 | -- |  |  |  |  |
| 8. Extraversion | 4.10 | 1.06 | .84 | .13** | -.18** | .06 | -.09 | -.28** | .05 | -.34** | -- |  |  |  |
| 9. Agreeableness | 4.35 | 0.94 | .80 | -.07 | .04 | -.23** | -.14** | -.29** | .36** | -.05 | .25** | -- |  |  |
| 10. Conscientiousness | 5.04 | 0.83 | .77 | .12* | -.15** | -.05 | -.00 | .09 | .25** | -.06 | .23** | .15** | -- |  |
| 11. Openness to Experience | 4.70 | 1.06 | .79 | -.37** | -0.04 | -.31** | -.06 | -.15** | .16** | -.01 | .03 | .12* | .09 | -- |
| 12. Left-Right Positioning  R | 4.19 | 1.98 | - | .51** | -.46** | .51** | -.08 | .07 | -.09 | -.22** | .15* | -.09 | .13* | -.21** |

*Note.* *N* = 409. * *p* < .05. ** *p* < .01. Variables 6 to 11 reflect the six HEXACO personality dimensions.

**APPENDIX G**

Overview of the individual trait measures used in Studies 4 and 5

**Right-Wing Authoritarianism**

1. Obedience and respect for authority are the most important virtues children should learn.

Young people sometimes get rebellious ideas, but as they grow up they ought to get over them and settle down.

3. Being kind to loafers or criminals will only encourage them to take advantage of our weakness, so it’s best to use a firm, tough hand when dealing with them.

4. It’s one thing to question and doubt someone during an election campaign, but once a man becomes the leader of our country we owe him our greatest support and loyalty.

5. In these troubled times laws have to be enforced without mercy, especially when dealing with agitators and revolutionaries who are stirring things.

6. One good way to teach certain people right from wrong is to give them a good stiff punishment when they get out of line.

7. Sex crimes, such as rape and attacks on children, deserve more than mere imprisonment; such criminals ought to be publicly whipped, or worse.

8. Our customs and national heritage are the things that have made us great, and certain people should be made to show greater respect for them.

9. In this complex world, you have to rely on experts and specialists.

10. Good leaders that are respected and supported by the people, have to be strict and demanding.

**Left-Wing Authoritarianism**

1. The rich should be stripped of their belongings and status.

2. Rich people should be forced to give up virtually all of their wealth.

3. If I could remake society, I would put people who currently have the most privilege at the very bottom.

4. Our country would be much better off if all of the rich people were at the bottom of the social ladder.

5. When the tables are turned on the oppressors at the top of society, I will enjoy watching them suffer the violence that they have inflicted on so many others.

6. Most rich Wall Street executives deserve to be thrown in prison.

7. Anyone who opposes gay marriage must be homophobic.

8. Deep down, just about all conservatives are racist, sexist, and homophobic.

9. Schools should be required by law to teach children about our country's history of racism, classism, sexism, and homophobia.

10. The "old-fashioned ways" and "old-fashioned values" need to be abolished.

11. People are truly worried about terrorism should shift their focus to the nutjobs on the far-right.

12. All political conservatives are fools.

13. Classroom discussions should be safe places that protect students from disturbing ideas.

14. University authorities are right to ban hateful speech from campus.

15. I should have the right not to be exposed to offensive views.

16. To succeed, a workplace must ensure that its employees feel safe from criticism.

17. I am in favor of allowing the government to shut down right-wing internet sites and blogs that promote nutty, hateful positions.

18. Colleges and universities that permit speaker with intolerant views should be publicly condemned.

**Social Dominance Orientation**

1. Some groups of people are simply not the equals of others.

2. Some people are just more worthy than others.

3. This country would be better off if we cared less about how equal all people were.

4. Some people are just more deserving than others.

5. It is not a problem if some people have more of a chance in life than others.

6. Some people are just inferior to others.

7. To get ahead in life, it is sometimes necessary to step on others.

8. One should strive for greater economic equality. (REC)

9. One should strive for more social equality. (REC)

10. “Equality” is an important value to me. (REC)

11. If people were treated more equally we would have fewer problems in this country. (REC)

12. In an ideal world, all nations would be equal. (REC)

13. We should try to treat one another as equals as much as possible. (REC)

14. It is important that we treat other countries as equals. (REC)

**Political cynicism**

1. People are very frequently manipulated by politicians.

2. Politicians are only interested in getting and maintaining power.

3. Politicians pretend to care more about people than they really do.

4. Our political leaders are prepared to lie to us whenever it suits their purposes.

5. If a politician sticks to his ideals and principles, he is unlikely to reach the top of his profession.

6. No man can hope to stay honest once he enters politics.

7. Almost all politicians will sell out their ideals or break their promises if it will increase their power.

8. All politicians are bad – some are just worse than others.

**Social cynicism**

1. I am often skeptical and cynical about people’s intentions.

2. People will tell lies to get ahead.

3. Most people are just out for themselves.

4. Many people take advantage of unselfish others.

5. People are out for what they can get.

6. Without being controlled, people don’t care about rules.

**HEXACO**

**Honesty-Humility**

1. I wouldn’t use flattery to get a raise or promotion at work, even if I thought it would succeed.

2. If I knew that I could never get caught, I would be willing to steal a million dollars. (REC)

3. Having a lot of money is not especially important to me.

4. I think that I am entitled to more respect than the average person is. (REC)

5. If I want something from someone, I will laugh at that person’s worst jokes. (REC)

6. I would never accept a bribe, even if it were very large.

7. I would get a lot of pleasure from owning expensive luxury goods. (REC)

8. I want people to know that I am an important person of high status. (REC)

9. I wouldn’t pretend to like someone just to get that person to do favors for me.

10. I’d be tempted to use counterfeit money, if I were sure I could get away with it. (REC)

**Emotionality**

1. I would feel afraid if I had to travel in bad weather conditions.

2. I sometimes can’t help worrying about little things.

3. When I suffer from a painful experience, I need someone to make me feel comfortable.

4. I feel like crying when I see other people crying.

5. When it comes to physical danger, I am very fearful.

6. I worry a lot less than most people do. (REC)

7. I can handle difficult situations without needing emotional support from anyone else. (REC)

8. I feel strong emotions when someone close to me is going away for a long time.

9. Even in an emergency I wouldn’t feel like panicking. (REC)

10. I remain unemotional even in situations where most people get very sentimental. (REC)

**Extraversion**

1. I feel reasonably satisfied with myself overall.

2. I rarely express my opinions in group meetings. (REC)

3. I prefer jobs that involve active social interaction to those that involve working alone.

4. On most days, I feel cheerful and optimistic.

5. I feel that I am an unpopular person. (REC)

6. In social situations, I’m usually the one who makes the first move.

7. The first thing that I always do in a new place is to make friends.

8. Most people are more upbeat and dynamic than I generally am. (REC)

9. I sometimes feel that I am a worthless person. (REC)

10. When I’m in a group of people, I’m often the one who speaks on behalf of the group.

**Agreeableness**

1. I rarely hold a grudge, even against people who have badly wronged me.

2. People sometimes tell me that I am too critical of others. (REC)

3. People sometimes tell me that I’m too stubborn. (REC)

4. People think of me as someone who has a quick temper. (REC)

5. My attitude toward people who have treated me badly is “forgive and forget.”

6. I tend to be lenient in judging other people.

7. I am usually quite flexible in my opinions when people disagree with me.

8. Most people tend to get angry more quickly than I do.

9. Even when people make a lot of mistakes, I rarely say anything negative.

10. When people tell me that I’m wrong, my first reaction is to argue with them. (REC)

**Conscientiousness**

1. I plan ahead and organize things, to avoid scrambling at the last minute.

2. I often push myself very hard when trying to achieve a goal.

3. When working on something, I don’t pay much attention to small details. (REC)

4. I make decisions based on the feeling of the moment rather than on careful thought. (REC)

5. When working, I sometimes have difficulties due to being disorganized.

6. I do only the minimum amount of work needed to get by. (REC)

7. I always try to be accurate in my work, even at the expense of time.

8. I make a lot of mistakes because I don’t think before I act. (REC)

9. People often call me a perfectionist.

10. I prefer to do whatever comes to mind, rather than stick to a plan. (REC)

**Openness to Experience**

1. I would be quite bored by a visit to an art gallery. (REC)

2. I’m interested in learning about the history and politics of other countries.

3. I would enjoy creating a work of art, such as a novel, a song, or a painting.

4. I think that paying attention to radical ideas is a waste of time. (REC)

5. If I had the opportunity, I would like to attend a classical music concert.

6. I’ve never really enjoyed looking through an encyclopedia. (REC)

7. People have often told me that I have a good imagination.

8. I like people who have unconventional views.

9. I don’t think of myself as the artistic or creative type. (REC)

10. I find it boring to discuss philosophy. (REC)

**Left-Right Positioning**

Many people use the terms “left” and “right” to denote different political attitudes. Here we have a scale that runs from left to right. When you think of your own political views where would you position yourself on this scale? (0 = left, 10 = right).

**APPENDIX H**

**TABLE H.1.** Overview of the 55 pairwise comparisons used in Study 5.

|  | Model A (citizens vs. government) | Model B  (citizens vs. government) |
| --- | --- | --- |
| Comparison 1 | 0-100 | 10-90 |
| Comparison 2 | 0-100 | 20-80 |
| Comparison 3 | 0-100 | 30-70 |
| Comparison 4 | 0-100 | 40-60 |
| Comparison 5 | 0-100 | 50-50 |
| Comparison 6 | 0-100 | 60-40 |
| Comparison 7 | 0-100 | 70-30 |
| Comparison 8 | 0-100 | 80-20 |
| Comparison 9 | 0-100 | 90-10 |
| Comparison 10 | 0-100 | 100-0 |
| Comparison 11 | 10-90 | 20-80 |
| Comparison 12 | 10-90 | 30-70 |
| Comparison 13 | 10-90 | 40-60 |
| Comparison 14 | 10-90 | 50-50 |
| Comparison 15 | 10-90 | 60-40 |
| Comparison 16 | 10-90 | 70-30 |
| Comparison 17 | 10-90 | 80-20 |
| Comparison 18 | 10-90 | 90-10 |
| Comparison 19 | 10-90 | 100-0 |
| Comparison 20 | 20-80 | 30-70 |
| Comparison 21 | 20-80 | 40-60 |
| Comparison 22 | 20-80 | 50-50 |
| Comparison 23 | 20-80 | 60-40 |
| Comparison 24 | 20-80 | 70-30 |
| Comparison 25 | 20-80 | 80-20 |
| Comparison 26 | 20-80 | 90-10 |
| Comparison 27 | 20-80 | 100-0 |
| Comparison 28 | 30-70 | 40-60 |
| Comparison 29 | 30-70 | 50-50 |
| Comparison 30 | 30-70 | 60-40 |
| Comparison 31 | 30-70 | 70-30 |

*Table H.1 continued*

|  | Model A (citizens vs. government) | Model B  (citizens vs. government) |
| --- | --- | --- |
| Comparison 32 | 30-70 | 80-20 |
| Comparison 33 | 30-70 | 90-10 |
| Comparison 34 | 30-70 | 100-0 |
| Comparison 35 | 40-60 | 50-50 |
| Comparison 36 | 40-60 | 60-40 |
| Comparison 37 | 40-60 | 70-30 |
| Comparison 38 | 40-60 | 80-20 |
| Comparison 39 | 40-60 | 90-10 |
| Comparison 40 | 40-60 | 100-0 |
| Comparison 41 | 50-50 | 60-40 |
| Comparison 42 | 50-50 | 70-30 |
| Comparison 43 | 50-50 | 80-20 |
| Comparison 44 | 50-50 | 90-10 |
| Comparison 45 | 50-50 | 100-0 |
| Comparison 46 | 60-40 | 70-30 |
| Comparison 47 | 60-40 | 80-20 |
| Comparison 48 | 60-40 | 90-10 |
| Comparison 49 | 60-40 | 100-0 |
| Comparison 50 | 70-30 | 80-20 |
| Comparison 51 | 70-30 | 90-10 |
| Comparison 52 | 70-30 | 100-0 |
| Comparison 53 | 80-20 | 90-10 |
| Comparison 54 | 80-20 | 100-0 |
| Comparison 55 | 90-10 | 100-0 |

**APPENDIX I**

Which decision-making model do you find most APPROPRIATE?

**Model A: Model B:**

- Citizens: 0% - Citizens: 10%
 - Government: 100% - Government: 90%

**FIGURE I.1.** Example of the presentation of a pairwise comparisons (Study 5).

**APPENDIX J**

**TABLE J.1.** The means, standard deviations, Cronbach’s alphas, and intercorrelations among the individual trait measures (Study 5).

| Variable | *M* | *SD* | α | 1. | 2. | 3. | 4. | 5. | 6. | 7. | 8. | 9. | 10. | 11. |
| --- | --- | --- | --- | --- | --- | --- | --- | --- | --- | --- | --- | --- | --- | --- |
| 1. Right-Wing Authoritarianism | 3.59 | 1.20 | .88 | -- |  |  |  |  |  |  |  |  |  |  |
| 2. Left-Wing Authoritarianism | 3.33 | 0.92 | .86 | -.20** | -- |  |  |  |  |  |  |  |  |  |
| 3. Social Dominance Orientation | 2.52 | 1.09 | .92 | .56** | -.34** | -- |  |  |  |  |  |  |  |  |
| 4. Political Cynicism | 5.00 | 1.04 | .86 | .03 | .27** | .06 | -- |  |  |  |  |  |  |  |
| 5. Social Cynicism | 4.81 | 0.97 | .84 | .25** | .12 | .24** | .46** | -- |  |  |  |  |  |  |
| 6. Honesty-Humility | 4.87 | 0.96 | .74 | -.05 | -.25** | -.18** | -.14* | -.20** | -- |  |  |  |  |  |
| 7. Emotionality | 4.31 | 1.01 | .80 | -.18** | .30** | -.22** | .17** | -.01 | -.10 | -- |  |  |  |  |
| 8. Extraversion | 4.11 | 1.01 | .81 | .18** | -.10 | -.03 | -.23** | -.23** | .16* | -.26** | -- |  |  |  |
| 9. Agreeableness | 4.25 | 0.96 | .80  20 | -.07 | -.08 | -.16* | -.20** | -.24** | .24** | -.18** | .36** | -- |  |  |
| 10. Conscientiousness | 5.10 | 0.84 | .77 | .10 | -.15* | -.05 | -.11 | -.04 | .27** | -.08 | .17** | .19** | -- |  |
| 11. Openness to Experience | 4.77 | 1.05 | .79 | -.29** | -.02 | -.27** | -.15* | -.13* | .17** | -.01 | .10 | .12 | .10 | -- |
| 12. Left-Right Positioning | 4.13 | 2.13 | - | .56** | -.50** | .50** | -.13* | .07 | .07 | -.25** | .15* | -.05 | .09 | -.20** |

*Note.* *N* = 240. * *p* < .05. ** *p* < .01. Variables 6 to 11 reflect the six HEXACO personality dimensions.
